# Stretchable Electronic Skins Realized by Confined In Situ Silver Patterning on Siloxane‐Modified Polyimide

**DOI:** 10.1002/advs.78699

**Published:** 2026-11-09

**Authors:** Jialun Yin, Yongjun Kang, Fangzhou Liu, Yifei Tong, Guofeng Tian, Shengli Qi, Dezhen Wu

**Affiliations:** ^1^ State Key Laboratory of Chemical Resource Engineering Beijing University of Chemical Technology Beijing China; ^2^ Changzhou Institute of Advanced Materials Beijing University of Chemical Technology Changzhou Jiangsu China

**Keywords:** confined metallization, electronic skin, in‐situin situ silver patterning, LC resonator, skin‐conformal electronics, stretchable polyimide

## Abstract

The rapid development of soft robotics and embodied intelligence demands electronic skins (E‐skins) that are both mechanically self‐adaptive and skin‐conformal. However, the rigid aromatic backbones of conventional polyimides limit stretchability and hinder conformal integration with deformable surfaces. Here, elastically stretchable polyimide (es‐PI) films are developed by incorporating aminopropyl‐terminated siloxane spacers, while a confined‐patterned in situ self‐metallization strategy enables direct fabrication of patterned silver circuits. By spatially confining Ag^+^ coordination and reduction within predefined regions, this strategy produces continuous silver nanolayers with well‐defined boundaries and robust metal–polymer interfaces, preserving electrical integrity during repeated deformation. The selected es‐PI‐0.5 substrate exhibits an estimated nominal elongation at break of approximately 96% and a tensile strength of approximately 25 MPa, while the corresponding metallized film retains electrical continuity during tensile deformation up to 50% strain. Device‐level feasibility is demonstrated through a strain‐dependent inductor–capacitor (LC) resonance response measured using a network analyzer and a separate smartphone‐assisted near‐field excitation experiment with rectified direct‐current (DC) output detected using a multimeter. This work establishes a materials–circuit integration approach for stretchable electronic skins and skin‐mounted sensing demonstrations.

## Graphical abstract

Stretchable polyimide substrates support directly patterned silver circuits for skin‐mounted motion‐detection demonstrations. Device‐level feasibility is further explored through strain‐dependent resonance measurements and a separate smartphone‐assisted near‐field excitation experiment with rectified DC output, illustrating the potential of the platform for deformable circuits and near‐field electronics.

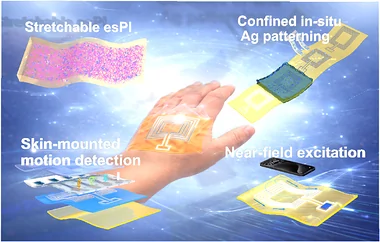

## 1 Introduction

Stretchable electronics have emerged as a transformative technology, enabling applications ranging from wearable health monitoring and soft robotics to human–machine interfaces and the Internet of Things [1, 2, 3, 4]. In particular, skin‐conformal devices that intimately interface with the human body have attracted significant attention, as they can accommodate the natural deformation of biological tissues while maintaining stable electronic functionality [5, 6, 7, 8]. The realization of such skin‐conformal systems imposes stringent requirements on substrate materials, which must simultaneously provide high stretchability to accommodate skin deformation, sufficient mechanical strength for practical handling, thermal stability to withstand device fabrication processes, and, crucially, the capability to support reliable electrical interconnects and sensing elements directly on their surfaces. For wireless devices operating in the near‐field communication (NFC) regime, these requirements become even more demanding: the substrate must not only serve as a passive mechanical support but also enable the integration of high‐quality‐factor antennas and sensing layers without compromising electrical performance [9, 10].

Among the various substrate candidates for flexible electronics, aromatic polyimides (PIs) have been widely adopted owing to their exceptional thermal stability, outstanding chemical resistance, excellent electrical insulation, and high mechanical strength [11, 12, 13]. These attributes make PIs the substrate of choice for commercial flexible printed circuit boards and high‐temperature electronic applications. However, the rigid aromatic backbones that confer these advantages also limit chain segmental mobility, which can constrain their ability to accommodate large tensile deformations. This mechanical constraint poses a challenge for skin‐conformal electronics, where substrates must accommodate tensile strains on the order of tens of percent associated with natural joint motion and skin deformation [6, 14, 15]. Beyond this mechanical limitation, conventional PIs present a second, equally critical challenge: their chemically inert surfaces. The imide rings and aromatic moieties dominating the PI structure offer few reactive sites for metal coordination or chemical bonding. As a result, the fabrication of high‐resolution conductive patterns on PI surfaces typically requires either adhesion‐promoting interlayers (such as Cr or Ti) or aggressive surface treatments that may degrade the underlying polymer [16, 17]. Even with such approaches, interfacial adhesion between metals and PI remains weak, often leading to delamination, cracking, or electrical failure under mechanical deformation. The mechanical rigidity and surface inertness of conventional PIs thus constitute a dual bottleneck that hinders their application in skin‐conformal wireless electronics, where both stretchability and reliable electrical patterning are indispensable.

To address the mechanical limitations of PIs, extensive efforts have been devoted to introducing flexible segments—such as aliphatic diamines, ether linkages, or siloxane chains—into the polymer backbone [18, 19, 20, 21, 22]. These molecular engineering strategies can effectively enhance chain mobility and increase elongation at break. However, they frequently come at the expense of thermal stability, as the flexible segments lower the glass transition temperature, and mechanical strength, as reduced chain rigidity decreases the tensile modulus. Achieving a substantial improvement in stretchability while simultaneously preserving the thermal and mechanical properties of pristine PIs remains a persistent trade‐off that is difficult to resolve at the molecular level [23]. In parallel, considerable efforts have been directed toward metallizing PI surfaces via physical vapor deposition, electroless plating, or surface grafting of catalytic species [24, 25, 26, 27]. While these approaches can generate conductive coatings, they typically suffer from one or more limitations, including restricted feature resolution due to mask constraints, poor interfacial adhesion that requires additional adhesion layers, complex multistep processing, or incompatibility with the mechanical deformation required for stretchable substrates. These considerations motivate the development of stretchable PI substrates that balance mechanical and thermal properties while supporting directly formed conductive patterns with well‐defined boundaries and resistance to interfacial failure under deformation.

To address these challenges for electronic skin (E‐skin) applications, a siloxane‐modified stretchable polyimide (es‐PI) is synthesized via copolymerization of 3,3′,4,4′‐biphenyltetracarboxylic dianhydride (BPDA), 4,4′‐oxydianiline (ODA), and aminopropyl‐terminated poly(dimethylsiloxane) (PDMS). Building on established approaches to intrinsically stretchable siloxane‐containing polyimides, [22, 28, 29, 30] BPDA/ODA is selected as a defined aromatic polyimide framework for balancing tensile strength, deformability, and thermal stability. Keeping the dianhydride unchanged while varying the diamine composition allows these property trade‐offs to be examined within a consistent copolymer series. The poly(amic acid) (PAA) precursor route also provides a processing platform for subsequent confined in situ silver patterning. The selected es‐PI‐0.5 substrate exhibits an estimated nominal elongation at break of approximately 96%, compared with approximately 9.5% for the unmodified BPDA/ODA PI prepared and tested in this study, while maintaining a tensile strength of approximately 25 MPa and a 5% weight‐loss temperature of 445°C. This composition is selected for its balance of mechanical and thermal properties rather than maximum elongation alone. In this design, siloxane incorporation tunes the mechanical properties of the PI substrate, while the PAA precursor provides carboxylic acid groups for Ag^+^ coordination. Spatial confinement of the coordination and reduction process enables conductive silver patterns to be formed in predefined regions. The resulting es‐PI‐0.5@Ag films exhibit well‐defined pattern boundaries, resistance to repeated tape removal, and retained electrical continuity under the tested deformations. These results demonstrate the feasibility of integrating a stretchable PI substrate with confined in situ silver patterning for skin‐mounted sensing applications. Since metallization was not compared across unmodified PI and different siloxane compositions under identical conditions, the present study does not establish a siloxane‐specific enhancement of metallization or interfacial adhesion. Molecular dynamics simulations show that siloxane incorporation increases the fractional free volume and decreases the mean‐square end‐to‐end distance of the simulated chains, while density functional theory calculations indicate that silver incorporation modifies the electronic structure and lowers the first‐step barrier along the calculated imidization pathway. Device‐level feasibility is explored through the strain‐dependent resonance response of an inductor–capacitor (LC) device and a separate smartphone‐assisted demonstration of near‐field excitation and rectified direct‐current (DC) output. These results demonstrate the integration of a mechanically balanced, stretchable polyimide substrate with directly patterned silver circuits for conformal electronic skin applications. Figure 1 summarizes the molecular design, confined in situ silver patterning strategy, and device‐level demonstrations of the es‐PI platform.

**FIGURE 1:**
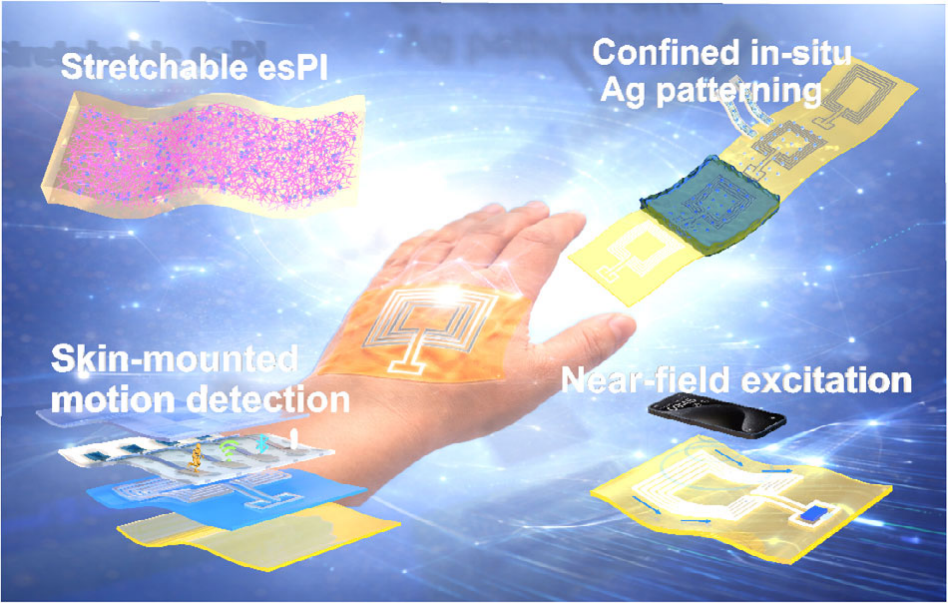
Overview of stretchable electronic skins realized by confined in situ silver patterning on siloxane‐modified polyimide. The es‐PI substrate supports directly patterned silver circuits for skin‐mounted motion‐detection demonstrations. Device‐level feasibility is explored through strain‐dependent resonance measurements and a separate smartphone‐assisted demonstration of near‐field excitation and rectified DC output. Insets illustrate the molecular design, confined silver patterning process, and device concepts.

## 2 Results and Discussion

### 2.1 Molecular Design and Mechanical Properties of Stretchable Polyimide

To address the intrinsic brittleness of conventional aromatic polyimides, a molecular‐level strategy was implemented by copolymerizing BPDA and ODA monomers with an aminopropyl‐terminated siloxane comonomer. The resulting siloxane‐modified polyimide (denoted as es‐PI) was designed to enhance chain mobility while preserving the thermal and mechanical properties of the parent PI. Figure 2a schematically illustrates the es‐PI film under uniaxial stretching, together with its molecular design. The incorporated siloxane segments act as flexible spacers, enabling reversible conformational rearrangement of polymer chains during stretching. The inset depicts the proposed chain evolution before and after tensile deformation, where the siloxane linkages accommodate applied strain without disrupting the imide‐rich hard domains. The chemical incorporation of siloxane moieties was confirmed by Fourier‐transform infrared (FTIR) spectroscopy. As shown in Figure 2b, the es‐PI film exhibits the characteristic absorption bands of the polyimide backbone, including symmetric and asymmetric C = O stretching vibrations and C–N bending vibrations of the imide ring, consistent with the formation of a fully cyclized polyimide structure [11]. In addition to these imide signatures, distinct peaks associated with the siloxane component are clearly observed, including characteristic Si–O–Si stretching and vibrational modes [31], confirming the successful incorporation of siloxane groups into the polymer network via copolymerization without disrupting the imidization process. The mechanical performance of es‐PI was systematically evaluated as a function of siloxane content, with detailed stress–strain curves provided in Figure S1 (Supporting Information) and key tensile parameters summarized in Figure 2c. The mechanical properties were evaluated using five independent specimens per composition, with the results reported as mean ± standard deviation (Figure 2c and Supplementary Table S2). Pristine BPDA/ODA‐type PI exhibits a tensile strength of 125.90 ± 3.28 MPa and an estimated nominal elongation at break of 9.51 ± 0.40%. Upon incorporation of siloxane segments, the tensile strength decreases to 78.57 ± 0.58 MPa for es‐PI‐0.1 and 63.26 ± 1.26 MPa for es‐PI‐0.2, while the corresponding elongation estimates increase to 18.88 ± 0.31% and 57.80 ± 2.59%, respectively. At an intermediate composition, es‐PI‐0.5 achieves a tensile strength of 25.10 ± 0.78 MPa and an estimated nominal elongation at break of 95.61 ± 3.71%. Further increasing the siloxane content to es‐PI‐0.8 increases the elongation estimate to 547.49 ± 59.26%, accompanied by a marked reduction in tensile strength to 3.65 ± 0.34 MPa. These results support the selection of es‐PI‐0.5 as a formulation balancing mechanical strength and deformability, with a mean elongation estimate approximately 10.1 times that of pristine PI. The elongation values were calculated after referencing each curve to its first recorded strain, using the original initial grip separation as the denominator. They represent nominal estimates subject to the zero‐reference assumption described in Section 4.5. The origin of this enhancement can be attributed to increased fractional free volume and altered chain conformation induced by siloxane segments, as further supported by molecular dynamics simulations presented later in this work. To benchmark the mechanical performance of es‐PI‐0.5, Figure 2d compares its tensile strength and elongation at break with those of reported poly(imide‐siloxane) films [29, 30] and commercial silicone elastomers, including Ecoflex 00–10, Dragon Skin 10 MEDIUM, and Dragon Skin 30. The plotted data show that es‐PI‐0.5 combines a tensile strength of approximately 25 MPa with an estimated nominal elongation at break of approximately 96%, providing higher tensile strength but lower extensibility than the selected silicone elastomers. Among the poly(imide‐siloxane) films shown, es‐PI‐0.5 exhibits greater elongation than Son‐PIS‐1, while retaining higher tensile strength than Choi‐PI‐3, Son‐PIS‐3, and Son‐PIS‐5. These comparisons highlight the balance between mechanical strength and extensibility achieved by es‐PI‐0.5, consistent with its selection as a stretchable substrate that retains useful load‐bearing capability. The literature and manufacturer data were obtained under different testing conditions, so this comparison serves as a contextual benchmark. The nanoindentation force–displacement response of es‐PI‐0.5 is provided in Figure S10 (Supporting Information). The incorporation of siloxane segments also significantly influences the thermal properties of the copolymers. Thermal stability was evaluated by thermogravimetric analysis (TGA), as shown in Figure S2 (Supporting Information). The pristine BPDA/ODA‐type PI exhibits a 5% weight loss temperature (Td5%) of 539°C. With increasing siloxane content, Td5% decreases to 494°C (es‐PI‐0.1), 465°C (es‐PI‐0.2), and 445°C (es‐PI‐0.5), while the residual weight at 800°C correspondingly decreases to 60.9%, 54.3%, 44.8%, and 31.4%, respectively. Notably, even for es‐PI‐0.5, Td5% remains well above 400°C, indicating that the copolymer retains sufficient thermal stability for flexible electronics applications. Dynamic mechanical analysis (Figure S3, Supporting Information) shows that the glass transition temperature (Tg) decreases from 282.1°C for pristine PI to 183.1°C for es‐PI‐0.5, accompanied by progressive broadening of the tan δ peak, suggesting increasingly heterogeneous segmental relaxation. Thermomechanical analysis (Figure S4, Supporting Information) further reveals that the coefficient of thermal expansion (CTE) increases from 45.5 ppm K^−^^1^ for pristine PI to 185.3 ppm K^−^^1^ for es‐PI‐0.5, demonstrating the influence of siloxane incorporation on the thermal expansion behavior of the films. The thermal properties of pristine PI and the es‐PI films are summarized in Supplementary Table S3 (Supporting Information). The environmental stability of the es‐PI films was evaluated by ultraviolet (UV) aging and simulated sweat immersion. The films exhibit only marginal changes in tensile strength and elongation at break after UV exposure at 60°C for 8 h (Figure S5, Supporting Information), while the FTIR spectrum of es‐PI‐0.5 remains essentially unchanged (Figure S6, Supporting Information). Following immersion in laboratory‐prepared simulated sweat at pH 4.7 ± 0.1 and room temperature for 48 h, es‐PI‐0.5 shows no appreciable changes in mass or dimensions (Figure S7, Supporting Information). These observations support the stability of the measured properties under the specific exposure conditions and durations examined. UV–vis spectroscopy (Figure S8, Supporting Information) shows a moderate decrease in transparency for es‐PI‐0.5 compared with pristine PI, which may be attributed to light scattering from microphase‐separated siloxane domains [32]. Water contact angle measurements (Figure S9, Supporting Information) reveal an increase from 55.6° for pristine PI to 85.1° for es‐PI‐0.5, likely arising from the surface enrichment of low‐surface‐energy siloxane segments. The mechanical compliance of es‐PI‐0.5 on human skin was further evaluated through finger bending tests. As shown in Figure 2e, the bending force increases slightly from 1.16 N (bare finger) to 1.41 N (with es‐PI‐0.5 attached), indicating that finger bending remained possible with a relatively small additional force in this demonstration. The film also withstands biaxial stretching, twisting, and weight loading without visible damage (Figure 2f). The es‐PI‐0.5 base film was subjected to 100 loading–unloading cycles at 90% strain (Figure 2g). Its overall length increased from 51.25 to 52.32 mm when measured 1 h after unloading, corresponding to an approximately 2.09% increase in overall specimen length. This observation indicates substantial dimensional recovery under the tested conditions, although the overall‐length measurement does not represent a gauge‐length‐based residual strain determination. The film followed the finger contour during the demonstrated bending motion (Figure 2h), illustrating its ability to accommodate a curved, moving surface. Skin adhesion strength and contact area were not quantified, and contact stability under prolonged movement or sweating remains to be evaluated. Finally, two separate forearm‐wearing tests were conducted by one of the authors, with continuous wearing durations of 6 and 12 h, respectively (Figure 2i). Visual inspection after film removal showed no visible redness following the 6 h test and slight redness following the 12 h test. These observations document the visible skin response of a single participant under the tested conditions and do not constitute a systematic assessment of skin compatibility.

**FIGURE 2:**
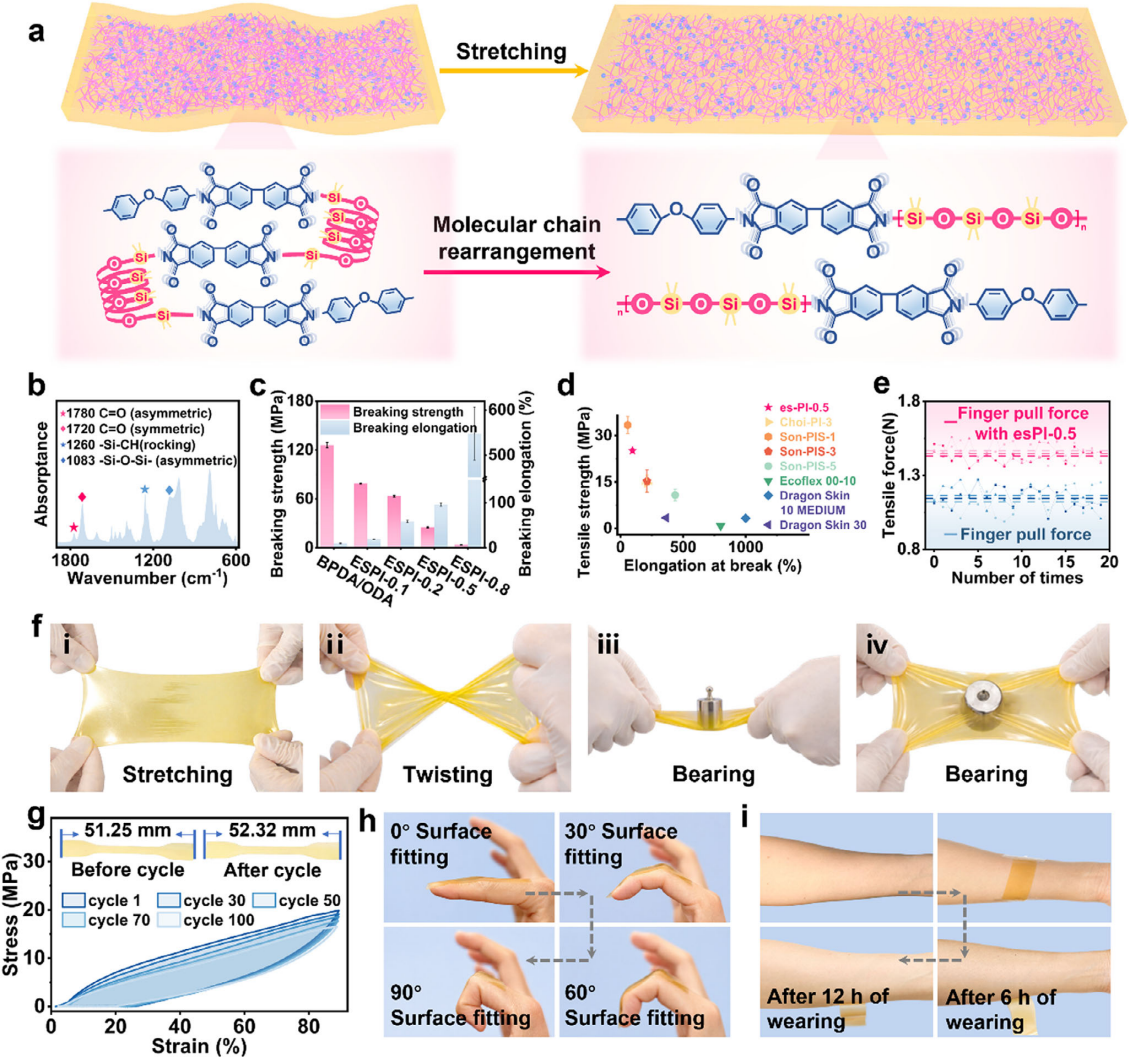
Molecular design, mechanical performance and skin‐attachment demonstrations of elastically stretchable polyimide (es‐PI). a, Schematic illustration of the molecular design of es‐PI and reversible chain rearrangement during uniaxial stretching. b, Fourier‐transform infrared (FTIR) spectra of pristine PI and es‐PI, showing characteristic imide and siloxane vibrations. c, Tensile strength and estimated nominal elongation at break of pristine PI and es‐PI films with different siloxane contents. Data are presented as mean ± standard deviation from five independent specimens per composition (n = 5). Elongation estimates were calculated using the first‐point reference procedure described in Section 4.5. d, Comparison of tensile strength and elongation at break of es‐PI‐0.5 with reported poly(imide‐siloxane) films and commercial silicone elastomers, using literature and manufacturer data obtained under different testing conditions [29, 30]. e, Finger bending force measured before and after attachment of the es‐PI‐0.5 film. f, Mechanical deformation of the es‐PI‐0.5 film under biaxial stretching, twisting and weight loading. g, Cyclic tensile stress‐strain curves of es‐PI‐0.5 at 90% strain for 100 cycles. Insets, optical images before and after cyclic stretching. h, Conformal attachment of the es‐PI‐0.5 film to a finger joint during bending. i, Photographs of the forearm skin after film removal following two separate continuous‐wearing tests of 6 and 12 h, conducted by the same participating author. No visible redness was observed after 6 h of wearing, whereas slight redness was observed after 12 h. The indicated times represent wearing durations, not post‐removal recovery intervals.

### 2.2 Confined‐Patterned In situ Self‐Metallization

To construct conductive patterns on the es‐PI substrate, a confined in situ self‐metallization strategy was developed using the ion‐exchange capability of the polyamic acid (PAA) precursor. Figure 3a schematically illustrates the fabrication process. Screen printing was used to define the PAA patterns, and an additional patterning demonstration was performed by pneumatically assisted printing using an HW‐DJS44 system connected to an air compressor. In both routes, the circuit geometry was defined at the precursor‐patterning stage. The patterned PAA film was then immersed in a 0.4 M AgNO_3_ solution, allowing Ag^+^ incorporation through interactions with carboxylate groups to form the PAA@Ag^+^ intermediate. Subsequent treatment with dimethylamine borane (DMAB) reduced the incorporated Ag^+^ to silver nanoparticles, yielding PAA@Ag. Thermal imidization converted the precursor into polyimide and promoted nanoparticle growth and coalescence to form the es‐PI‐0.5@Ag conductive patterns. The preparation procedures are described in Section 4.3 and Supplementary Method S2.

**FIGURE 3:**
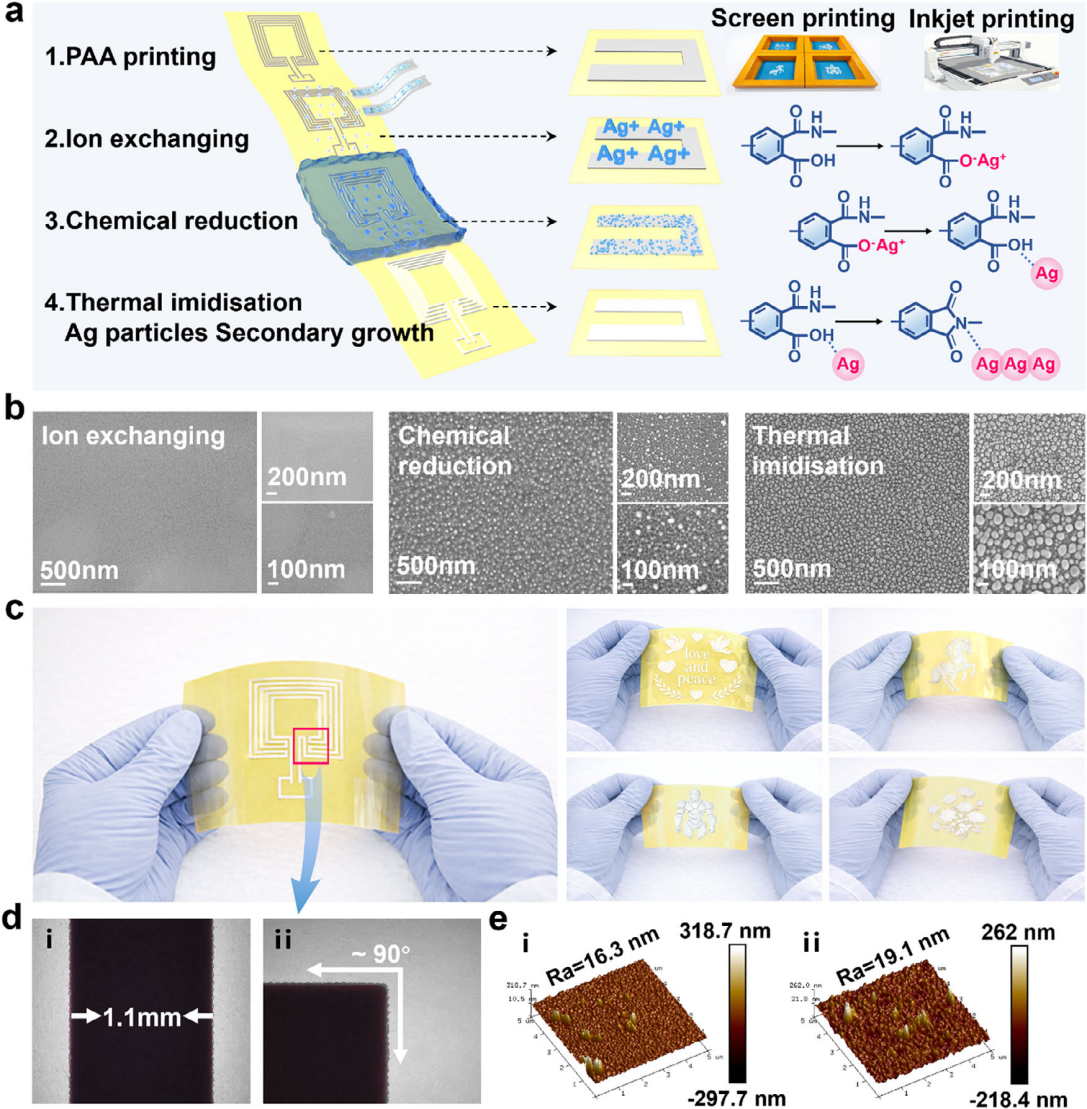
Confined in situ silver patterning on elastically stretchable polyimide. a, Schematic illustration of PAA precursor patterning by screen printing or pneumatically assisted printing, followed by Ag^+^ ion exchange, chemical reduction, and thermal imidization. The corresponding chemical transformations are shown alongside. b, Scanning electron microscopy (SEM) images showing the morphological evolution of the patterned surface after ion exchange, reduction, and thermal imidization. c, Representative conductive patterns on es‐PI‐0.5. The first image shows a sample prepared by pneumatically assisted printing of the PAA precursor using an HW‐DJS44 system connected to an air compressor, followed by metallization. d, Optical microscopy images of patterned lines and right‐angle features, showing well‐defined boundaries, a demonstrated linewidth of approximately 1.1 mm, and near‐right‐angle corners. e, Atomic force microscopy (AFM) topography and surface roughness (R_a) of pristine es‐PI‐0.5 and es‐PI‐0.5@Ag.

The morphological evolution at each stage was examined by scanning electron microscopy (SEM; Figure 3b). The ion‐exchanged film exhibits a smooth surface without observable particle formation, consistent with Ag^+^ being retained in an ionic state within the PAA matrix. After reduction, uniformly distributed Ag nanoparticles with diameters of 20–50 nm appear across the surface, confirming effective nucleation. Following thermal imidization, the particle size increases to ∼100 nm with reduced inter‐particle spacing [33], forming a nearly continuous conductive network. This evolution may be attributed to enhanced atomic mobility of silver at elevated temperatures, which promotes particle coalescence. Notably, the thermal imidization step serves a dual function, completing PI formation while simultaneously driving consolidation of the conductive network. The chemical interactions underlying ion exchange were confirmed by FTIR spectroscopy (Figure S11, Supporting Information), where the characteristic band at 1403 cm^−^^1^ in pristine PAA shifts significantly after Ag^+^ loading, indicative of coordination with carboxylate groups. X‐ray photoelectron spectroscopy (XPS) measurements were performed at different processing stages to characterize silver incorporation and the final metallized film. The survey spectra of the PAA precursor before and after Ag^+^ ion exchange show the appearance of silver signals following ion exchange (Figure S12‐1, Supporting Information), supporting silver incorporation into the precursor. Separately, the Ag 3d spectrum was collected from the final es‐PI‐0.5@Ag film after chemical reduction and thermal imidization (Figure S12‐2, Supporting Information). The survey and high‐resolution spectra therefore characterize distinct sample states and are interpreted separately. Ion‐exchange kinetics were evaluated by varying immersion time (Figure S13, Supporting Information). Below 40 min, nanoparticle density and uniformity progressively increase. At 40 min, a dense and homogeneous distribution is achieved, whereas longer times lead to surface defects likely caused by excessive swelling of the uncured precursor. Water contact angle measurements (Figure S14, Supporting Information) show a similar trend, increasing and then plateauing within 40 min, indicating saturation of the ion‐exchange process. Representative conductive patterns, including rectangular spiral inductors and decorative motifs, are shown in Figure 3c. The first image shows a metallized pattern prepared by pneumatically assisted printing of the PAA precursor using an HW‐DJS44 system connected to an air compressor, followed by ion exchange, chemical reduction, and thermal imidization. This sample demonstrates the feasibility of applying the metallization sequence to a precursor pattern produced by this printing route. Optical microscopy (Figure 3d) shows a demonstrated linewidth of approximately 1.1 mm, well‐defined edges, and near‐right‐angle corners. These observations support spatially confined pattern formation at the demonstrated dimensions; the minimum achievable linewidth and edge‐placement accuracy were not determined. Atomic force microscopy (AFM; Figure 3e) shows a modest increase in the measured surface roughness after metallization, from R_a = 16.3 nm for pristine es‐PI‐0.5 to 19.1 nm for es‐PI‐0.5@Ag. These results characterize the exposed surface topography and indicate that metallization produces only a limited change in the measured roughness. The AFM measurements do not determine the depth distribution of silver within the polymer or quantify nanoparticle embedding.

### 2.3 Electromechanical Performance of Patterned Silver Circuits

The electromechanical stability of conductive patterns under deformation is critical for stretchable electronic skin systems. Figure 4a presents optical images of the es‐PI‐0.5@Ag film before and after stretching, showing a macroscopically continuous silver pattern without obvious cracks at the scale of observation. The accompanying schematic illustrates a proposed interfacial configuration involving partial embedding of silver particles at the polymer surface. Such a configuration could contribute to interfacial anchoring through mechanical interlocking, although the extent of embedding and its contribution to adhesion were not directly determined.

**FIGURE 4:**
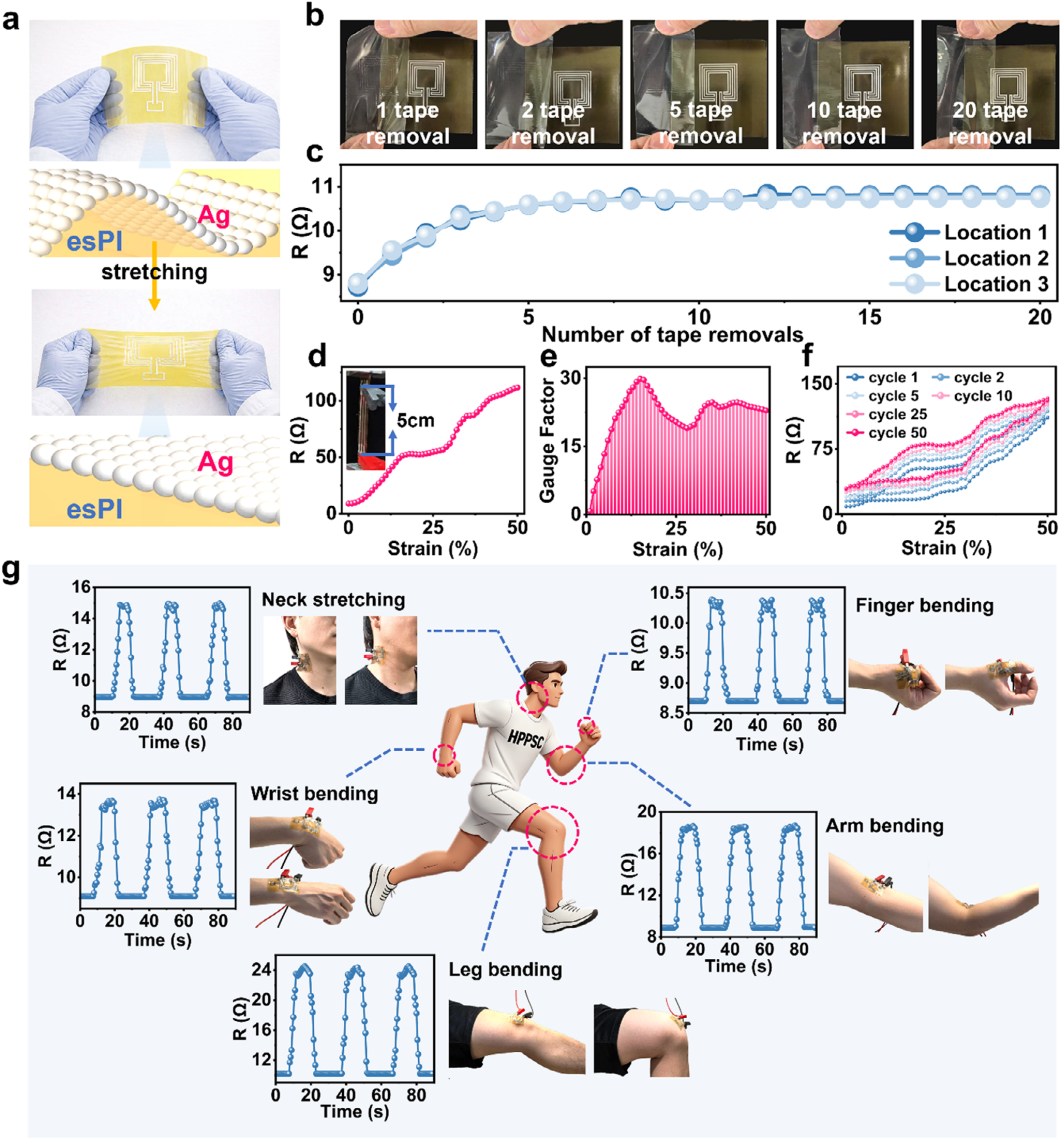
Electromechanical performance of the patterned silver network on elastically stretchable polyimide. a, Optical images of es‐PI‐0.5@Ag before and after stretching, together with a schematic of a proposed metal–polymer interfacial configuration involving partial embedding of silver particles. b, Tape‐removal test for qualitative assessment of the adhesion of the patterned silver layer. c, Resistance evolution during repeated tape removal at three measurement locations (zone 1–3) on the same specimen, rather than three independent specimens. d, Resistance change of es‐PI‐0.5@Ag under uniaxial stretching to 50% strain. e, Gauge factor as a function of applied strain. f, Resistance variation during 50 stretching–releasing cycles at 50% strain. g, Resistance responses of the skin‐conformal es‐PI‐0.5@Ag device attached to the neck, elbow, wrist, finger, and knee during human motion.

The adhesion of the patterned silver layer was qualitatively assessed using repeated tape‐removal tests. Figure 4b shows optical images of the adhesive tape after 1, 2, 5, 10, and 20 removal cycles. After the first removal, a clear silver imprint is observed on the tape; this imprint becomes progressively weaker after the second cycle and is reduced to trace levels after five cycles. After 20 cycles, no obvious silver residue is visible on the tape in the optical images, suggesting that loosely bound surface particles are progressively removed during the initial cycles. Figure 4c summarizes the corresponding resistance evolution at three locations on the same specimen. The resistance increases modestly from an initial value of 8.89 Ω to approximately 10.5 Ω after five cycles and subsequently stabilizes at approximately 10.7 Ω, supporting the retention of an electrically connected silver network during repeated tape removal. Together, these observations support the adhesion and electrical integrity of the silver layer under the tested conditions. Because the tape was removed manually without quantitative control of peeling angle or speed, this test provides a qualitative assessment of resistance to repeated tape removal and does not quantify interfacial adhesion strength.

The electromechanical response under uniaxial tension is shown in Figure 4d, where the measured resistance increases monotonically from approximately 9 Ω to 117 Ω at 50% nominal strain, while maintaining a continuous signal without interruption. The corresponding gauge factor (GF) (Figure 4e) reaches approximately 30 in the low‐strain regime (<15%) and approaches approximately 24 at higher strains (up to 50%), demonstrating pronounced strain sensitivity over the tested range. Cyclic electromechanical performance was evaluated over 50 stretching–releasing cycles at a maximum nominal strain of 50% (Figure 4f). Figure S15 (Supporting Information) presents the normalized resistance–strain curves for cycles 1, 2, 5, 10, 25, and 50, calculated as ΔR/R_0_ = (R − R_0_)/R_0_ using the initial resistance of 8.9 Ω as a common reference. The separation between the loading and unloading branches and the upward shift of the unloaded baseline indicate electrical hysteresis and baseline drift. The resistance measured 1 min after unloading following the 50th cycle was 29.04 Ω, corresponding to R_after/R_0_ ≈ 3.26 and ΔR/R_0_ ≈ 2.26 (226%). Despite this drift, the cyclic response remained continuous without signal dropout, demonstrating retained electrical continuity over the tested cycles. SEM images of the same specimen after cycling (Figure S16, Supporting Information) show an interconnected silver nanoparticle network without evident large‐scale cracking or delamination in the observed regions. The resistance increase may reflect local rearrangement of the conductive network and incomplete recovery of interparticle contacts; possible changes in contact resistance also cannot be separated by the two‐wire measurements. These results support continued conduction during repeated deformation, while the observed drift and hysteresis should be considered for quantitative strain sensing. Recovery beyond the 1 min observation interval remains to be evaluated.

To demonstrate the potential of es‐PI‐0.5@Ag for skin‐mounted motion monitoring, the film was attached to different body locations, including the neck, wrist, elbow, finger, and knee (Figure 4g). Three consecutive motion–relaxation cycles were recorded at each location, showing similar resistance‐response patterns within each short measurement sequence. The measured resistance increased during movement and decreased upon relaxation, indicating that the patterned silver circuits responded to deformation at the attachment sites. The peak resistance values ranged from approximately 10 Ω at the finger to 24 Ω at the knee. These differences may reflect variations in local deformation and attachment conditions and should not be interpreted as a calibrated comparison of strain across body locations. Upon release, the resistance returned toward its initial level, demonstrating a recoverable electrical response during the illustrated motions. These results provide an initial demonstration of motion detection using es‐PI‐0.5@Ag attached to curved skin surfaces. Extended cycling durability and the stability of skin contact during prolonged movement and sweating remain to be evaluated.

### 2.4 Molecular Insights Into Substrate Deformability and Confined Self‐Metallization

To elucidate the molecular‐level mechanisms underlying the experimentally observed mechanical behavior of the es‐PI system and the role of Ag^+^ in the metallization process, molecular dynamics (MD) simulations and density functional theory (DFT) calculations were performed. The pronounced difference in stretchability between BPDA/ODA PI and es‐PI‐0.5 (Figure 2c) motivated an investigation into the molecular origin of the enhanced chain mobility. Figure 5a presents the fractional free volume distributions of the two systems. The unmodified PI exhibits a fractional free volume of 37.6%, whereas es‐PI‐0.5 shows a higher value of 44.3%, indicating that siloxane incorporation introduces additional free volume within the polymer matrix that may facilitate segmental motion under external deformation. Complementarily, the mean‐square end‐to‐end distances, ⟨Ree^2^⟩ (Figure 5b), are 2431 Å^2^ for BPDA/ODA PI and 826.58 Å^2^ for es‐PI‐0.5. The lower value for es‐PI‐0.5 indicates a more compact equilibrium chain conformation in the simulated model. Together with the increase in fractional free volume, this result is consistent with greater scope for conformational rearrangement during deformation and provides molecular‐level support for the experimentally observed enhancement in stretchability. Together, the increased free volume and enhanced conformational flexibility are consistent with the greater deformability of es‐PI‐0.5, whose mean estimated nominal elongation at break is approximately 10.1 times that of pristine PI. The role of Ag^+^ coordination in modulating the electronic structure and intermolecular interactions of the PAA precursor was further investigated using DFT calculations. Electrostatic potential maps (Figure 5c) reveal that Ag^+^ coordination induces pronounced charge redistribution along the PAA backbone. The carboxylate oxygen involved in Ag^+^ binding becomes more electron‐rich, while polarization of the amide N–H bond is weakened, indicating a substantial perturbation of the local electronic environment. Further insight is provided by the energy‐level diagrams of the highest occupied molecular orbital (HOMO) and lowest unoccupied molecular orbital (LUMO) (Figure 5d). The calculated gap decreases from 3.88 eV for PAA to 2.13 eV for the silver‐containing model, indicating a change in the frontier orbital energy separation upon silver incorporation. The energetics of proton removal from the amide N–H site were further evaluated by calculating the gas‐phase deprotonation energy, defined as Δ*E_deprot_*  =  *E*(*A* ^−^) +  *E*(*H* ^+^) − *E*(*AH*), where AH and A^−^ denote the protonated model and its corresponding deprotonated species, respectively. The calculated values are −1.62 kcal mol^−^^1^ for PAA and −2.76 kcal mol^−^^1^ for the silver‐containing model (Figure 5e). The lower value for the latter indicates more favorable deprotonation energetics within the calculated models. This comparison concerns electronic‐energy differences and does not directly establish a reduction in the activation barrier for proton transfer. Figure 5f compares the van der Waals surface penetration maps of PAA and PAA@Ag^+^. The PAA@Ag^+^ system exhibits significantly greater intermolecular surface penetration, suggesting strengthened intermolecular interactions upon Ag^+^ incorporation. This enhanced cohesion is further reflected in solubility tests: pristine PAA fully dissolves in DMF within 30 min, whereas PAA@Ag^+^ remains intact even after 48 h of immersion (Figure S17, Supporting Information), indicating Ag^+^‐mediated intermolecular crosslinking that stabilizes the precursor and benefits subsequent pattern formation. The influence of silver incorporation on the imidization pathway was examined by transition‐state calculations (Figure 5g). The calculated pathways for both models contain two transition states separated by an intermediate. Each transition state exhibits a single imaginary frequency corresponding to the intended reaction coordinate. Forward and reverse intrinsic reaction coordinate (IRC) calculations were also performed for ten steps in each direction to examine the local paths around these structures (Figure S18, Supporting Information).

**FIGURE 5:**
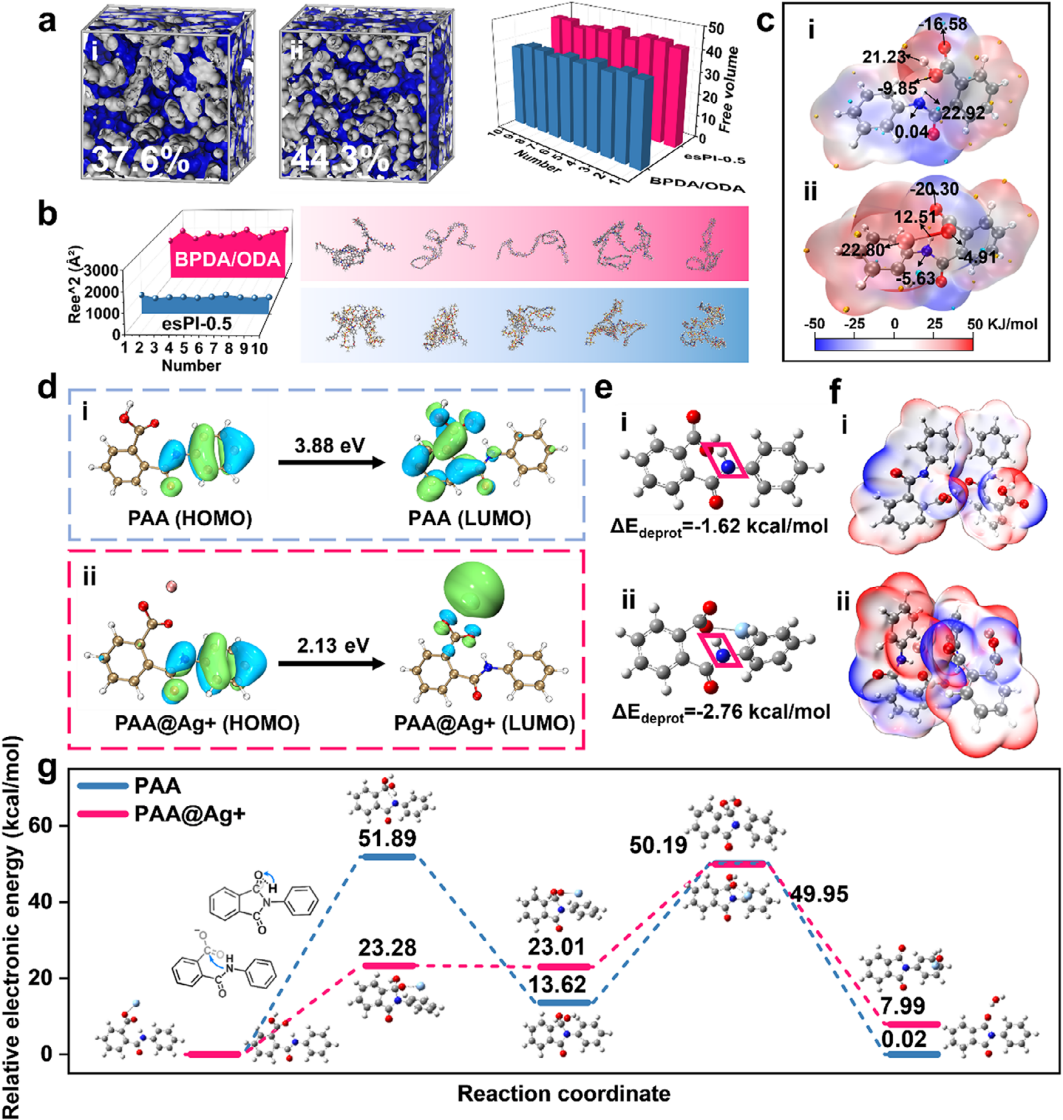
Molecular‐level analysis of the stretchable polyimide substrate and silver‐containing precursor models. a, Fractional free volume distributions of BPDA/ODA PI and es‐PI‐0.5. b, Mean‐square end‐to‐end distances, ⟨Ree^2^⟩, and representative chain conformations of BPDA/ODA PI and es‐PI‐0.5. c, Electrostatic potential maps of PAA and the silver‐containing model. d, Energy‐level diagrams of the highest occupied molecular orbital (HOMO) and lowest unoccupied molecular orbital (LUMO), with energies expressed in eV. e, Calculated gas‐phase deprotonation energies at the amide N–H site. f, Intermolecular surface interaction maps of the two models. g, Calculated imidization pathways expressed as relative electronic energies, with the initial structure of each pathway taken as its respective reference. Zero‐point vibrational energy and thermal corrections are not included.

Figure 5g presents relative electronic energies, referenced to the initial structure of each respective pathway, without zero‐point vibrational energy or thermal corrections. The corresponding calculated electronic energies and relative energies are listed in Supplementary Table S4 (Supporting Information). The first‐step electronic‐energy barrier decreases from 51.89 kcal mol^−^^1^ for PAA to 23.28 kcal mol^−^^1^ for the silver‐containing model. The second‐step barrier, referenced to the intermediate, decreases from 36.56 to 26.95 kcal mol^−^^1^. However, the highest transition‐state energies relative to the respective initial structures are 51.89 and 49.95 kcal mol^−^^1^. Thus, the calculations indicate a substantial reduction in the first‐step barrier and a redistribution of the energy profile, rather than an equally large decrease in the energy requirement of the complete pathway. Collectively, these results support a role for siloxane segments in modifying the conformational characteristics of the substrate and for silver incorporation in altering the electronic structure and calculated cyclization energetics of the precursor. The MD and DFT calculations address different aspects of the material system. The MD results provide molecular‐level insight into changes in chain conformation and fractional free volume associated with siloxane incorporation. The DFT calculations, however, employ a simplified PAA model without siloxane segments and its corresponding silver‐containing model. The calculated changes in electronic structure and the lower first‐step imidization barrier therefore apply to these model structures and do not establish a siloxane‐specific enhancement of silver coordination or patterning. Their relevance to the complete es‐PI‐0.5 precursor remains qualitative, and a direct comparison of metallization across different substrate compositions is required to determine whether siloxane incorporation affects pattern formation or interfacial adhesion.

### 2.5 LC Resonance and Smartphone‐Assisted Near‐Field Demonstrations

To demonstrate the potential utility of the es‐PI‐0.5@Ag platform in skin‐conformal electronics, an LC‐based device was constructed. Figure 6a schematically illustrates the device configuration. The spiral silver coil is directly patterned onto the es‐PI‐0.5 substrate via the confined‐patterned in situ growth strategy described in Section 2.2. The device incorporates PEDOT:PSS and a fixed capacitor, while a BAT54 Schottky diode enables rectification in the smartphone‐assisted demonstration. Figure 6b illustrates the concept of near‐field excitation using an NFC‐enabled smartphone. When the smartphone is brought close to the device with NFC activated, the alternating magnetic field induces an electrical response in the silver coil, and the rectified DC output is detected using a multimeter. As shown in Figure 6c, a DC output remained detectable at a separation distance of approximately 0.9 cm using a Redmi K70 smartphone. The smartphone application was used only to activate NFC operation; it did not receive or display data from the device. This observation supports the feasibility of integrating the patterned silver coil into a circuit for near‐field excitation and rectification. The demonstration was qualitative, without recorded output‐voltage values, and the tested separation does not establish a maximum operating range or quantitative power‐transfer performance.

**FIGURE 6:**
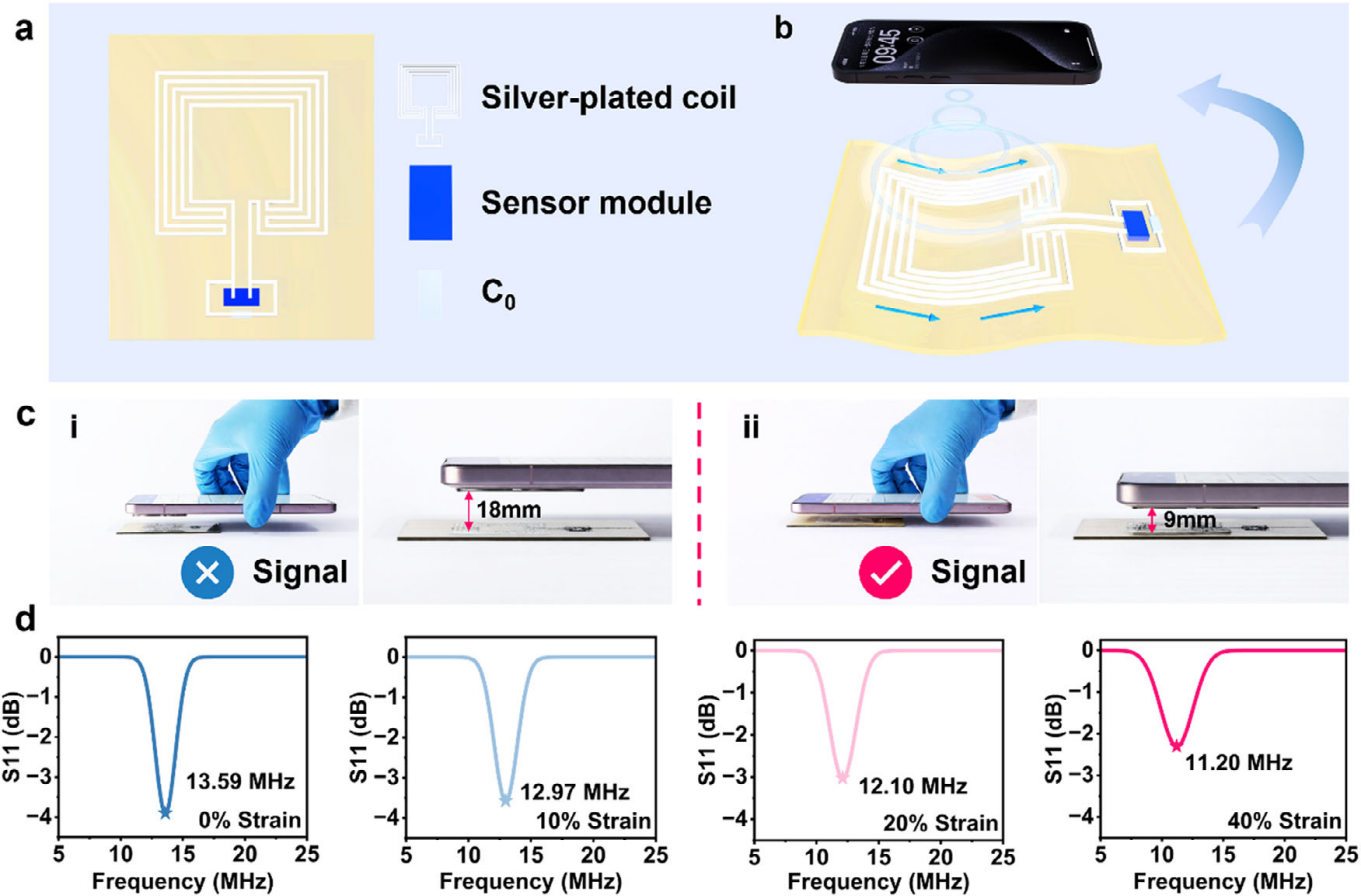
LC resonance and smartphone‐assisted near‐field demonstrations using the es‐PI‐0.5@Ag platform. a, Schematic illustration of an inductor–capacitor (LC) device incorporating a patterned silver spiral coil, a poly(3,4‐ethylenedioxythiophene):poly(styrenesulfonate) (PEDOT:PSS) layer, and a fixed capacitor. b, Conceptual illustration of near‐field excitation using an NFC‐enabled smartphone. c, Qualitative demonstration of smartphone‐assisted excitation at the indicated phone–device separations. “Signal” denotes detection of the rectified direct‐current (DC) output using a multimeter; the smartphone served as the excitation source and did not receive or display data from the device. d, S₁₁ reflection spectra measured separately using a network analyzer at overall tensile strains of 0%, 10%, 20%, and 40%.

The strain‐dependent resonance response was separately characterized using a PNA‐N5244A network analyzer while the device was subjected to overall tensile deformation. Figure 6d presents the S₁₁ spectra at applied strains of 0%, 10%, 20%, and 40%. In the unstrained state (0%), a distinct resonance dip is observed at 13.59 MHz with a minimum S₁₁ of approximately −3.9 dB. Upon stretching to 10%, the resonance shifts to 12.97 MHz, with a minimum of approximately −3.5 dB. At 20% strain, it further shifts to 12.10 MHz with a minimum of approximately −3.0 dB, and at 40% strain, it reaches 11.20 MHz with a minimum of approximately −2.4 dB. The monotonic decrease in resonance frequency demonstrates a measurable response to the applied deformation. This frequency shift reflects changes in the effective electrical parameters of the device during stretching; however, the individual contributions of coil deformation and the PEDOT:PSS component were not independently resolved. A progressive broadening and shallowing of the resonance dip are also observed with increasing strain. These changes may be associated in part with deformation‐induced resistive losses, consistent with the resistance increase observed in Figure 4d, although the quality factor and individual loss contributions were not quantitatively evaluated. Together, the strain‐dependent S₁₁ response and the separate smartphone‐assisted rectification demonstration illustrate the potential of the es‐PI‐0.5@Ag platform for deformable resonant circuits and near‐field electronic applications. These results provide preliminary device‐level validation, while quantitative smartphone‐based strain readout and continuous wireless motion monitoring remain to be established.

## 3 Conclusion

In summary, we demonstrate a strategy for the fabrication of self‐adaptive and conformal electronic skins based on elastically stretchable polyimide (es‐PI) and confined in situ silver patterning. The siloxane‐modified polyimide provides a stretchable and thermally stable substrate for electronic skin integration, while confined in situ self‐metallization enables the direct formation of patterned silver circuits on its surface. The resulting platform combines substrate stretchability and mechanical strength with conductive patterns that retain electrical continuity under the tested deformations. Preliminary skin‐mounted measurements demonstrate motion‐dependent electrical responses, while longer‐term cycling durability and skin‐interface stability remain to be established. Device‐level feasibility is further illustrated by strain‐dependent resonance measurements using a network analyzer and a separate smartphone‐assisted demonstration of near‐field excitation and rectified DC output. These preliminary results support the potential of the es‐PI‐0.5@Ag platform for deformable resonant circuits and near‐field electronics. More broadly, this work provides a materials–patterning approach that integrates stretchable polyimide substrates with directly formed conductive circuits, supporting further development of electronic skins for soft robotics, human–machine interfaces, and wearable electronics.

## 4 Experimental Section/Methods

### 4.1 Materials

3,3′,4,4′‐Biphenyltetracarboxylic dianhydride (BPDA, 99%), 4,4′‐oxydianiline (ODA, 99%), aminopropyl‐terminated poly(dimethylsiloxane) (PDMS; nominal molecular weight of approximately 1000 g mol^−^^1^), dimethylacetamide (DMAc, 99%), and poly(3,4‐ethylenedioxythiophene):poly(styrenesulfonate) (PEDOT:PSS) dispersion (1.3 wt.%) were purchased from Macklin and used as received. Silver nitrate (AgNO_3_, 99%) and dimethylamine borane (DMAB, 98%) were obtained from Aladdin (Shanghai, China).

### 4.2 Synthesis of Stretchable Polyimide (es‐PI)

Stretchable polyimide (es‐PI) was synthesized through a conventional two‐step poly(amic acid) (PAA) route. For es‐PI‐0.5, ODA (0.529 g, 2.64 mmol) and aminopropyl‐terminated PDMS (2.640 g, approximately 2.64 mmol) were dissolved in DMAc (18.9 g) and stirred for 30 min. BPDA (1.553 g, 5.28 mmol) was subsequently added, and the reaction was conducted in an ice‐water bath for 2 h to obtain a PAA precursor solution with a solid content of approximately 20 wt.%. The solid content was calculated as the combined mass of ODA, PDMS, and BPDA divided by the total mass of these monomers and DMAc. The precursor was cast and dried at 60°C, followed by thermal imidization in air at 300°C for 2 h to afford the es‐PI film. In the designation es‐PI‐x, x represents the molar fraction of PDMS in the total diamine feed, defined as n(PDMS)/[n(ODA) + n(PDMS)]. The PDMS molar amount was estimated using the supplier‐reported nominal molecular weight of approximately 1000 g mol^−^^1^. Thus, es‐PI‐0.5 contains equal molar amounts of ODA and PDMS, with the total diamine feed equimolar to BPDA. The feed compositions of the other samples are listed in Supplementary Table S1.

### 4.3 Fabrication of Confined In Situ Silver Patterns

Patterned conductive silver layers were fabricated through precursor patterning followed by ion exchange, chemical reduction, and thermal imidization. For screen printing, the PAA precursor solution was deposited onto the es‐PI‐0.5 substrate through a patterned screen stencil and dried at 60°C for 30 min. An additional patterning demonstration was performed using an HW‐DJS44 system connected to an air compressor to print the PAA precursor; the resulting metallized sample is shown in the first image of Figure 3c. This route is referred to as pneumatically assisted printing. The two precursor‐patterning procedures are described separately in Supplementary Method S2. Specific operating settings for the HW‐DJS44 demonstration are not reported, limiting reproducibility of this example.

The patterned PAA films were immersed in 0.4 M AgNO_3_ solution for 40 min to form the ion‐exchanged intermediate (PAA@Ag^+^), rinsed with deionized water, and subsequently treated with 0.005 M DMAB solution for 15 min to generate silver nanoparticles (PAA@Ag). Thermal imidization at 300°C for 2 h converted PAA into polyimide and promoted nanoparticle coalescence, yielding the final es‐PI‐0.5@Ag conductive patterns.

### 4.4 Fabrication of Devices for LC Resonance and Near‐Field Demonstrations

Spiral silver coils were patterned directly onto es‐PI‐0.5 substrates using the confined in situ silver patterning procedure described in Section 4.3. The components used in the device demonstrations included PEDOT:PSS and a fixed capacitor (C_0_). The smartphone‐assisted rectification demonstration additionally incorporated a BAT54 Schottky diode, and no NFC integrated circuit was used. The capacitor value and detailed component interconnections could not be verified from the available records. Measurement procedures and the scope of the device demonstrations are described in Supplementary Method S5.

### 4.5 Characterization

Fourier transform infrared (FTIR) spectra were recorded using a Nicolet Nexus 670 spectrometer over 600–3000 cm^−^^1^. Raman spectra were collected on a Horiba LabRAM HR Evolution spectrometer with a 532 nm excitation laser. Surface morphology was characterized by scanning electron microscopy (SEM, ZEISS GeminiSEM 300) and transmission electron microscopy (TEM, FEI Talos F200X G2). X‐ray photoelectron spectroscopy (XPS) was performed on a Thermo Scientific K‐Alpha spectrometer equipped with a monochromatic Al Kα source.

Tensile tests were conducted at room temperature using a universal testing machine (SANS CMT4101). The polymer substrates were approximately 40 µm thick, and the metallized specimens had a total thickness of approximately 42 µm. Dog‐bone‐shaped specimens had a narrow section approximately 4.5 mm wide and 22 mm long and were mounted with an initial grip separation of 20 mm. Monotonic tensile tests to fracture were performed at a crosshead speed of 5 mm min^−^^1^. Engineering stress (σ) and engineering strain (ε) were calculated as

$$\sigma =\frac{F}{A}$$

$$\varepsilon =\frac{\Delta x}{L_{0}}$$

where Δx is the crosshead displacement and L_0_ is the initial grip separation. No preload was applied. For the composition‐dependent tensile analysis, each curve was retrospectively referenced to its first recorded strain. The estimated nominal elongation at break was calculated as

$$\;\varepsilon_{est}=\varepsilon_{rec}\;-\varepsilon_{first}$$

where ε_rec_ is the recorded nominal strain immediately before the terminal fracture‐related stress drop and ε_first_ is the first recorded strain. The original initial grip separation was retained as the denominator. Strain values were multiplied by 100 when expressed as percentages. Stress values were unchanged by this adjustment.

For cyclic tensile testing, the loading and unloading crosshead speeds were both 0.5 mm min^−^^1^. The es‐PI‐0.5 substrate in Figure 2g was tested for 100 cycles at a maximum nominal strain of 90%, whereas the metallized specimen in Figure 4f was tested for 50 cycles at a maximum nominal strain of 50%. For the overall‐length comparison in Figure 2g, the specimen was allowed to recover for 1 h after the final unloading step before its total length was measured. The length change was referenced to the initial total specimen length of 51.25 mm, rather than the initial grip separation. Further details of the mechanical testing and strain estimation are provided in Supplementary Method S4.

Dynamic mechanical analysis (DMA, TA Q800) was carried out in tensile mode at 1 Hz with a heating rate of 3°C min^−^^1^. The loss factor was calculated as

$$\;\tan\delta =\frac{E^{\prime \prime }}{E^{\prime }}$$

where *E′* and *E″* denote the storage and loss moduli, respectively. The glass transition temperature (T_g_) was determined from the maximum of the tan δ curve.

Thermogravimetric analysis (TGA) was performed under nitrogen at a heating rate of 10°C min^−^^1^. The decomposition temperature (T_d5%_) was defined as the temperature corresponding to 5 wt.% mass loss.

Thermomechanical analysis (TMA) was conducted at 5°C min^−^^1^. The coefficient of thermal expansion (CTE) was calculated according to

$$CTE=\frac{1}{L_{0}}\;\frac{\Delta L}{\Delta T}$$

where *ΔL* is the dimensional change, *L_0_* is the initial specimen length, and *ΔT* is the corresponding temperature interval.

Atomic force microscopy (AFM, Bruker Dimension ICON) was operated in tapping mode for surface morphology and in PeakForce quantitative nanomechanical mapping mode for mechanical characterization.

Static water contact angles were measured using 3 µL deionized water droplets. UV‐vis transmission spectra were recorded over 300–800 nm. Environmental stability was evaluated by UV aging (340 nm, 0.7 W m^−^^2^, 60°C, 8 h) and immersion in simulated sweat solution for 48 h.

The electrical measurements in Figure 4 were performed using a Keithley DMM6500 digital multimeter in a two‐wire configuration, with one lead connected to each end of a rectangular silver‐coated region approximately 30 mm long and 1.1 mm wide. The directly recorded quantity was resistance R in ohms. No conversion to sheet resistance or correction based on the dimensions of the deformed specimen was applied. During tensile testing, resistance was recorded as a function of time and related to nominal strain using the testing‐machine motion and initial grip separation. The measured resistance response includes contributions from changes in specimen geometry and the conductive network and may also include contributions from lead and contact resistances associated with the two‐wire configuration. The pointwise gauge factor was calculated as

$$GF=\frac{\left[\frac{R\;-\;R_{0}}{R_{0}}\right]}{\varepsilon }\;,\varepsilon \;>\;0$$

where R_0_ is the resistance immediately before the tensile loading shown in Figure 4d and ε is the dimensionless nominal strain (e.g., ε = 0.5 at 50% strain). This quantity is the normalized resistance change divided by the applied strain, rather than the local derivative of the response curve. For Figure 4f, the resistance of 29.04 Ω was recorded 1 min after unloading following the 50th cycle.

### 4.6 Molecular Dynamics Simulations

Molecular dynamics (MD) simulations were performed using the Forcite module in Materials Studio with the COMPASS II force field. Amorphous cells containing five polymer chains with a degree of polymerization of 20 were constructed for pristine BPDA/ODA PI and es‐PI‐0.5. After geometry optimization, each model was equilibrated by annealing between 300 and 600 K under the constant particle number, volume, and temperature (NVT) ensemble for 500 ps, followed by a 2 ns simulation in the constant particle number, pressure, and temperature (NPT) ensemble at 298 K and 1 atm. Fractional free volume was calculated using the Connolly surface method with a probe radius of 1.0 Å. The mean‐square end‐to‐end distance was calculated as

$$\;\langle Ree^{2}\rangle ={\left(r_{end}\;-\;r_{start}\right)}^{2}$$

where r_end_ and r_start_ are the position vectors of the two chain ends. Values were averaged over the final 500 ps of the equilibrated trajectories and are reported in Å^2^.

### 4.7 Density Functional Theory Calculations

Density functional theory (DFT) calculations were performed using Gaussian 16. Geometry optimizations employed B3LYP/6‐31G(d) for the PAA models and B3LYP/def2SVPP for all atoms in the silver‐containing models. Single‐point electronic energies were subsequently calculated at the B3LYP/def2TZVP level. Ag was treated using the effective core potentials associated with the def2 basis sets, replacing 28 core electrons. All calculations were performed in the gas phase without an implicit solvent model. The neutral PAA (C₁_4_H₁₁NO_3_) and silver‐containing (C₁_4_H₁_0_AgNO_3_) models were assigned a total charge of zero and a singlet multiplicity. The corresponding deprotonated species were assigned a charge of −1 and a singlet multiplicity, while H^+^ was assigned a charge of +1 and a multiplicity of 1. These simplified models do not contain siloxane segments.

Electrostatic potential distributions, frontier orbital energy levels, and gas‐phase deprotonation energies were evaluated using the optimized structures. The deprotonation energy at the amide N–H site was defined as

$$\Delta E_{deprot}=E\left(A^{-}\right)+E\left(H^{+}\right)-\;E\left(AH\right)$$

where AH denotes the neutral model before proton removal and *A* ^−^ denotes the corresponding deprotonated species. This quantity is an electronic‐energy difference and is distinct from a hydrogen‐bond interaction energy or a homolytic N–H bond dissociation energy.

Each transition state exhibited a single imaginary frequency corresponding to the intended reaction coordinate. Intrinsic reaction coordinate (IRC) calculations were performed in both directions at the respective geometry‐optimization levels, with ten steps in each direction. The trajectories terminated at the specified step limit and describe local paths around the transition states; convergence to endpoint minima was not established. Reaction‐energy profiles were constructed from the B3LYP/def2TZVP single‐point electronic energies without zero‐point vibrational energy or thermal corrections. Relative energies were referenced to the initial structure of each respective pathway, and each elementary‐step barrier was calculated relative to its preceding minimum. Energy differences were converted using 1 Hartree = 627.509474 kcal mol^−^^1^. Additional computational details are provided in Supplementary Method S7.

### 4.8 Statistical Analysis

For the composition‐dependent tensile results in Figure 2c and Supplementary Table S2, five independent specimens were included for each composition (n = 5). Tensile strength was determined as the maximum engineering stress of each individual stress–strain curve. The estimated nominal elongation at break was calculated separately for each specimen by subtracting its first recorded strain from the strain immediately before the fracture‐related stress drop, as described in Section 4.5 and Supplementary Method S4. The original initial grip separation was retained as the denominator, and stress values were unchanged by this adjustment. Arithmetic means and sample standard deviations, calculated using n − 1 in the variance denominator, were then obtained from the five specimen‐level values. All five specimens were included in this analysis, without exclusion based on high or low measured values. The reported properties were not extracted from an averaged stress–strain curve. The standard deviations describe specimen‐to‐specimen variability and do not include systematic uncertainty associated with the assumed strain reference or crosshead‐based strain measurement.

The electrical results in Figure 4 are based on directly recorded two‐wire resistance measurements, without conversion to sheet resistance. Derived quantities were calculated as follows. The pointwise gauge factor in Figure 4e was calculated as

$$GF=\frac{\left[\frac{R\;-\;R_{0}}{R_{0}}\right]}{\varepsilon }\;,\varepsilon \;>\;0$$

where R_0_ is the resistance before the tensile loading in Figure 4d and ε is dimensionless nominal strain. The normalized resistance changes in Figure S15 were calculated as

$$\frac{\Delta R}{R^{0}}=\left(R-R_{0}\right)/R_{0}$$

using the resistance before the first cycle, R₀ = 8.9 Ω, as a fixed reference for all displayed cycles. No cycle‐specific baseline resetting or additional vertical shifting was applied to these normalized curves.

The three measurement locations labelled zone 1–3 in Figure 4c correspond to different positions on the same specimen. The three consecutive motion–relaxation cycles at each body location in Figure 4g represent repeated responses within each measurement sequence. Measurement locations and repeated cycles were not treated as independent specimens. Thermal, spectroscopic, and microscopy results are presented descriptively, without inferential statistical comparisons.

### 4.9 Ethical Statement

The human‐involving demonstrations were conducted through non‐invasive self‐testing by one of the authors. No external volunteers were recruited, and no human tissues or bodily fluids were collected. No formal ethics committee approval or documented exemption was obtained for these self‐tests; therefore, no ethics approval or exemption reference number is available. Written informed consent was obtained from the participating author and retained, covering participation in the experiments and publication of the associated photographs and data. The simulated sweat solution was prepared from laboratory reagents and did not contain human‐derived biological materials.

## Author Contributions

**Jialun Yin**: writing – original draft, conceptualization, methodology, validation, visualization, software, data curation, investigation, writing – review and editing. **Yongjun Kang**: validation, formal analysis. **Fangzhou Liu**: software, data curation. **Yifei Tong**: software, data curation. **Guofeng Tian**: funding acquisition. **Shengli Qi**: writing – review and editing, funding acquisition, project administration, supervision, resources. **Dezhen Wu**: funding acquisition.

## Conflicts of Interest

The authors declare no conflicts of interest.

## Supporting information




**Supporting File**: advs78699‐sup‐0001‐SuppMat.docx.

## Data Availability

The data supporting the findings of this study are available from the corresponding author upon reasonable request.

## References

[1] J. A. Rogers , T. Someya , and Y. Huang , “Materials and Mechanics for Stretchable Electronics,” Science 327, no. 5973 (2010): 1603–1607, 10.1126/science.1182383. 20339064

[2] X. Wang , Z. Liu , and T. Zhang , “Flexible Sensing Electronics for Wearable/Attachable Health Monitoring,” Small 13, no. 25 (2017): 1602790, 10.1002/smll.201602790. 28306196

[3] W. Heng , S. Solomon , and W. Gao , “Flexible Electronics and Devices as Human–Machine Interfaces for Medical Robotics,” Advanced Materials 34, no. 16 (2022): 2107902, 10.1002/adma.202107902. PMC9035141 34897836

[4] K. K. Kim , Y. Suh , and S. H. Ko , “Smart Stretchable Electronics for Advanced Human–Machine Interface,” Advanced Intelligent Systems 3, 2 (2021): 2000157, 10.1002/aisy.202000157.

[5] C. Xu , S. A. Solomon , and W. Gao , “Artificial Intelligence‐Powered Electronic Skin,” Nature machine intelligence 5, no. 12 (2023): 1344–1355, 10.1038/s42256-023-00760-z. PMC10868719 38370145

[6] X. Liu , H. Li , M. Tao , et al., “Organic Flexible Electronics for Innovative Applications in Electronic Skin,” Advanced Materials Technologies 10, no. 3 (2025): 2400661, 10.1002/admt.202400661.

[7] H. Zhang , J. Hong , J. Zhu , et al., “Humanoid Electronic‐Skin Technology for the Era of Artificial Intelligence of Things,” Matter 8, no. 5 (2025): 102136, 10.1016/j.matt.2025.102136.

[8] Y. Lee , X. Tian , J. Park , et al., “Rapidly Self‐Healing Electronic Skin for Machine Learning–assisted Physiological and Movement Evaluation,” Science Advances 11, no. 7 (2025): ads1301, 10.1126/sciadv.ads1301. PMC11818020 39937914

[9] Y. Hu , F. Yang , L. Xu , Y. Wang , C. Zhang , and C. Song , “Flexible near‐Field Communication in Wearable Electronics: Antenna Design, Measurements, and System Demonstration,” IEEE Journal of Selected Areas in Sensors 3 (2025): 23–32, 10.1109/JSAS.2025.3635540.

[10] Z. Cao , P. Chen , Z. Ma , et al., “Near‐Field Communication Sensors,” Sensors 19, no. 18 (2019): 3947, 10.3390/s19183947. PMC6767079 31547400

[11] Y. Kang , D. Lin , B. Liu , et al., “In Situ Engineering of a 3D Branched Polyimide/Silver Interface Layer with Dual Ion‐Electron Conductivity for Superior Structural Integrity and Long‐Cycle Stability of Silicon Anodes,” Advanced Functional Materials 35, no. 44 (2025): 2504861, 10.1002/adfm.202504861.

[12] H. Ohya , V. V. Kudryavsev , and S. I. Semenova , Polyimide Membranes: Applications, Fabrications and Properties (Routledge, 2022), 10.1201/9780203742969.

[13] V. Daghigh , H. Daghigh , and R. Harrison , “High‐Temperature Polyimide Composites—A Review on Polyimide Types, Manufacturing, and Mechanical and Thermal Behavior,” Journal of Composites Science 9, no. 10 (2025): 526, 10.3390/jcs9100526.

[14] X. Lü , J. Yang , L. Qi , W. Bao , L. Zhao , and R. Chen , “High Sensitivity Flexible Electronic Skin Based on Graphene Film,” Sensors 19, no. 4 (2019): 794, 10.3390/s19040794. PMC6412976 30781388

[15] R. Guo , Y. Bao , X. Zheng , et al., “Organohydrogel Based Electronic Skin Reinforced by Dual‐Mode Conduction and Hierarchical Collagen Fibers Skeleton,” Advanced Science 12, no. 6 (2025): 2412934, 10.1002/advs.202412934. PMC11809366 39679908

[16] P.‐S. He , D.‐P. Tran , T.‐Y. Kuo , et al., “High‐Bonding‐Strength Polyimide Films Achieved via Thermal Management and Surface Activation,” Nanomaterials 13, no. 9 (2023): 1575, 10.3390/nano13091575. PMC10180403 37177120

[17] C. Yan , J. Li , Y. Dai , et al., “Fabrication of High‐Performance Ald‐Al_2_o_3_/Sio_2_ Nanolaminate Coating for Atomic Oxygen Erosion Resistance on Polyimide,” Surface and Coatings Technology 502 (2025): 131960, 10.1016/j.surfcoat.2025.131960.

[18] Y. Zhuang , J. G. Seong , and Y. M. Lee , “Polyimides Containing Aliphatic/Alicyclic Segments in the Main Chains,” Progress in Polymer Science 92 (2019): 35–88, 10.1016/j.progpolymsci.2019.01.004.

[19] S. Hicyilmaz and A. C. Bedeloglu , “Applications of Polyimide Coatings: a Review,” SN Applied Sciences 3, no. 3 (2021): 363, 10.1007/s42452-021-04362-5.

[20] A. Ghosh , S. Kumar Sen , S. Banerjee , and B. Voit , “Solubility Improvements in Aromatic Polyimides by Macromolecular Engineering,” RSC advances 2, no. 14 (2012): 5900–5926, 10.1039/c2ra20175e.

[21] S. Mo , L. Zhai , Y. Liu , et al., “Siloxane‐Containing Polyimide Films with High Heat Resistance and Low Dielectric Constant,” Iet Nanodielectrics 7, no. 1 (2024): 33–45, 10.1049/nde2.12064.

[22] H. Qi , X. Wang , T. Zhu , J. Li , L. Xiong , and F. Liu , “Low Dielectric Poly (Imide Siloxane) Films Enabled by a Well‐Defined Disiloxane‐Linked Alkyl Diamine,” ACS omega 4, no. 26 (2019): 22143–22151, 10.1021/acsomega.9b03302. PMC6933767 31891096

[23] D.‐J. Liaw , K.‐L. Wang , Y.‐C. Huang , K.‐R. Lee , J.‐Y. Lai , and C.‐S. Ha , “Advanced Polyimide Materials: Syntheses, Physical Properties and Applications,” Progress in Polymer Science 37, no. 7 (2012): 907–974, 10.1016/j.progpolymsci.2012.02.005.

[24] P. Augustyn , P. Rytlewski , K. Moraczewski , and A. Mazurkiewicz , “A Review on the Direct Electroplating of Polymeric Materials,” Journal of Materials Science 56, no. 27 (2021): 14881–14899, 10.1007/s10853-021-06246-w.

[25] L. Li , G. Yan , J. Wu , X. Yu , Q. Guo , and E. Kang , “Electroless Plating of Copper on Polyimide Films Modified by Surface‐Initiated Atom‐Transfer Radical Polymerization of 4‐Vinylpyridine,” Applied Surface Science 254, no. 22 (2008): 7331–7335, 10.1016/j.apsusc.2008.05.327.

[26] T.‐J. Liu , C.‐H. Chen , P.‐Y. Wu , C.‐H. Lin , and C.‐M. Chen , “Efficient and Adhesiveless Metallization of Flexible Polyimide by Functional Grafting of Carboxylic Acid Groups,” Langmuir 35, no. 22 (2019): 7212–7221, 10.1021/acs.langmuir.9b00354. 31083950

[27] P.‐Y. Wu , C.‐H. Lin , and C.‐M. Chen , “Study of Surface Metallization of Polyimide Film and Interfacial Characterization,” Metals 7, no. 6 (2017): 189, 10.3390/met7060189.

[28] L.‐C. Hsu , C.‐C. Shih , H.‐C. Hsieh , et al., “Intrinsically Stretchable, Solution‐Processable Functional Poly(siloxane‐imide)s for Stretchable Resistive Memory Applications,” Polymer Chemistry 9 (2018): 5145–5154, 10.1039/C8PY01283K.

[29] J. Y. Choi , K. N. Nam , S. W. Jin , et al., “Preparation and Properties of Poly(imide‐siloxane) Copolymer Composite Films with Micro‐Al2O3 Particles,” Applied Sciences 9, no. 3 (2019): 548, 10.3390/app9030548.

[30] Y. P. Son , D. Choi , J. Lee , et al., “Eco‐Friendly Fabrication of Sandwich‐Structured Poly(imide‐siloxane)/Silver Nanowire Composite Films for Stretchable Strain Sensors,” RSC Advances 15 (2025): 19645–19655, 10.1039/D5RA01386K. PMC12153423 40503321

[31] Z. Zhang , Z. Zhang , S. Yue , X. Jiang , and J. Hu , “Performance Characteristics of Silicone Rubber for Use in Acidic Environments,” Polymers 15, no. 17 (2023): 3598, 10.3390/polym15173598. PMC10489667 37688224

[32] C.‐K. Ku and Y.‐D. Lee , “Microphase Separation in Amorphous Poly (Imide Siloxane) Segmented Copolymers,” Polymer 48, no. 12 (2007): 3565–3573, 10.1016/j.polymer.2007.04.028.

[33] T. W. Hansen , A. T. DeLaRiva , S. R. Challa , and A. K. Datye , “Sintering of Catalytic Nanoparticles: Particle Migration or Ostwald Ripening?,” Accounts of chemical research 46, no. 8 (2013): 1720–1730, 10.1021/ar3002427. 23634641

