# Supplementary material for: Stretchable Electronic Skins Realized by Confined In Situ Silver Patterning on Siloxane‐Modified Polyimide
Source: Adv Sci (Weinh). 2026 Nov 9:e78699. Online ahead of print. doi: 10.1002/advs.78699 (PMC13748897; doi:10.1002/advs.78699)
Supplement: Supplementary file 1 [file ADVS-9999-e78699-s001.docx]

Supporting Information

**Stretchable Electronic Skins Realized by Confined In-situ Silver Patterning on Siloxane-Modified Polyimide**

*Jialun Yin, Yongjun Kang, Fangzhou Liu, Yifei Tong, Guofeng Tian, Shengli Qi*, Dezhen Wu*

**Table of Contents**

[**Supplementary Methods	3**](#_Toc235117097)

[**Supplementary Notes	9**](#_Toc235117098)

[**Supplementary Figures	13**](#_Toc235117099)

[**Supplementary Tables	24**](#_Toc235117100)

**Supplementary Methods**

**S1. Preparation of simulated sweat solution and immersion testing**

Polyimide is abbreviated PI. The sample labelled PI is the unmodified polyimide prepared from 3,3′,4,4′-biphenyltetracarboxylic dianhydride (BPDA) and 4,4′-oxydianiline (ODA). The siloxane-modified stretchable polyimides are denoted es-PI and contain aminopropyl-terminated poly(dimethylsiloxane) (PDMS) as the siloxane comonomer. In es-PI-x, x denotes the nominal molar fraction of PDMS in the total diamine feed. Poly(amic acid) (PAA) denotes the precursor before imidization, and es-PI-0.5@Ag denotes the metallized es-PI-0.5 film.

A laboratory-prepared simulated sweat solution was used for the immersion test. Sodium chloride (5.0 g L⁻¹), lactic acid (1.0 g L⁻¹), and urea (1.0 g L⁻¹) were dissolved in deionized water under magnetic stirring. The pH was adjusted to 4.7 ± 0.1 using aqueous ammonia, and the final volume was adjusted with deionized water.

The es-PI film specimens were immersed in freshly prepared solution at room temperature for 48 h. At predetermined intervals, the specimens were removed, gently wiped with filter paper to remove surface liquid, and weighed immediately. Dimensional changes were determined by measuring the film length and width using a digital caliper.

The solution was prepared entirely from laboratory reagents. No human-derived sweat, tissues, or bodily fluids were used.

**S2. Precursor patterning by screen printing and pneumatically assisted printing**

For the screen-printing route, a stainless-steel screen stencil containing the desired circuit patterns was fabricated using a commercial photolithographic process. The PAA precursor solution was applied to the stencil and printed onto the es-PI-0.5 substrate using a polyurethane squeegee. The screen-printed films were dried at 60 °C for 30 min to remove most of the solvent while retaining the PAA precursor state. These processing conditions refer to the screen-printing route.

For the additional pneumatically assisted printing demonstration, the PAA precursor was patterned using an HW-DJS44 system connected to an air compressor. The resulting precursor pattern was subsequently subjected to the ion-exchange, chemical-reduction, and thermal-imidization procedure described in Section 4.3 of the main text. The corresponding metallized sample is shown in the first image of Figure 3c. Specific printing parameters, including dispensing pressure and printing speed, are not reported. This example supports the feasibility of precursor patterning followed by metallization at the demonstrated dimensions, while reproducibility of the printing step is limited by the absence of these settings. No systematic optimization of printing resolution or quantitative process-yield assessment is reported.

**S3. Ion-exchange and reduction process**

The patterned PAA films were immersed in a 0.4 M AgNO₃ aqueous solution at room temperature for 5, 10, 20, 40, or 60 min to investigate the ion-exchange kinetics. After ion exchange, the samples were rinsed thoroughly with deionized water to remove physically adsorbed Ag⁺ ions.

The ion-exchanged films were subsequently immersed in a freshly prepared 0.005 M dimethylamine borane (DMAB) aqueous solution for 15 min at room temperature. After reduction, the samples were rinsed again with deionized water and dried under ambient conditions.

Thermal imidization was then carried out in air at 300 °C for 2 h, simultaneously converting PAA into polyimide and promoting coalescence of silver nanoparticles into a continuous conductive layer.

**S4. Mechanical testing**

Dog-bone-shaped specimens were prepared according to ASTM D882. Tensile tests were performed using a universal testing machine equipped with a 100 N load cell at room temperature with a crosshead speed of 5 mm min^-^¹.

The polymer substrates were approximately 40 μm thick, and the metallized specimens had a total thickness of approximately 42 μm. Dog-bone-shaped specimens had a narrow section approximately 4.5 mm wide and 22 mm long and were mounted with an initial grip separation of 20 mm.

Engineering stress was calculated as

$$\sigma=\frac{F}{A_{0}}$$

where F is the applied force and A₀ is the initial cross-sectional area. Recorded nominal strain was calculated as

$$\varepsilon_{rec} = \Delta x/L₀$$

where Δx is the crosshead displacement and L₀ is the initial grip separation. No preload was applied.

For the composition-dependent tensile analysis, each curve was retrospectively referenced to its first recorded strain. The estimated nominal elongation at break was calculated as

$$\varepsilon_{est}=\varepsilon_{rec}-\varepsilon_{first}$$

where ε_rec_ is the recorded nominal strain immediately before the terminal fracture-related stress drop and ε_first_ is the first recorded strain. The original initial grip separation was retained as the denominator, and stress values were unchanged. Strain values were multiplied by 100 when expressed as percentages.

This procedure assumes that the first recorded point approximates the strain reference after initial specimen straightening. However, the first recorded points exhibited nonzero stress, and no earlier force–displacement records or independently measured straightening displacements were available to validate this assumption. The reported elongations are therefore estimates based on the first recorded point, and the adjustment should not be interpreted as a validated correction for initial slack. Because the available initial loading records do not support reliable determination of Young’s modulus, this parameter is not reported.

Five independent specimens were included for each composition (n = 5). Tensile strength was determined as the maximum engineering stress of each individual curve. The elongation estimate was calculated separately for each specimen before determining the arithmetic mean and sample standard deviation. No specimens were excluded on the basis of high or low measured results. The standard deviations describe specimen-to-specimen variability and do not include systematic uncertainty associated with the assumed zero reference or crosshead-based strain measurement.

For cyclic tensile testing, the loading and unloading crosshead speeds were both 0.5 mm min⁻¹. The es-PI-0.5 substrate shown in Figure 2g was subjected to 100 cycles at a maximum nominal strain of 90%. After the final unloading step, the specimen was allowed to recover for 1 h before its overall length was measured. The overall specimen length increased from 51.25 mm before cycling to 52.32 mm after this recovery interval, corresponding to an overall length increase of approximately 2.09%. This quantity was calculated using the original overall specimen length and does not represent residual strain measured within the initial 20 mm grip separation.

**S5. Inductor–capacitor (LC) resonance measurements and smartphone-assisted near-field demonstration**

Strain-dependent S₁₁ spectra were measured using a PNA-N5244A network analyzer while the device was subjected to overall tensile strains of 0%, 10%, 20%, and 40%. The available records did not establish whether the analyzer was connected directly to the device or to an external coupling coil, and the associated measurement geometry could not be verified. The spectra are therefore reported as strain-dependent resonance responses, without claiming a verified contactless wireless measurement configuration.

In a separate near-field excitation demonstration, a Redmi K70 smartphone was used with near-field communication (NFC) activated through the VFC tool application. The circuit incorporated the patterned silver coil, poly(3,4-ethylenedioxythiophene):poly(styrenesulfonate) (PEDOT:PSS), a fixed capacitor (C₀), and a BAT54 Schottky diode. A multimeter was used to detect the rectified DC output. The smartphone application served only to activate NFC operation and did not receive or display data from the circuit.

A rectified direct-current (DC) output was detectable at a phone–device separation of approximately 0.9 cm. Numerical output-voltage values and the multimeter model were not recorded. This observation is presented as a qualitative demonstration of near-field excitation and rectification at the tested separation; it does not establish a maximum operating range, quantitative power-transfer performance, or smartphone-based strain readout. The detailed circuit interconnections and capacitor value could not be verified from the available records.

**S6. Molecular dynamics simulations**

Molecular dynamics (MD) simulations were performed using the Forcite module in Materials Studio with the COMPASS II force field.

Amorphous cells containing five polymer chains with a degree of polymerization of 20 were constructed for both BPDA/ODA polyimide and es-PI-0.5. Geometry optimization was followed by five annealing cycles between 300 and 600 K under the constant particle number, volume, and temperature (NVT) ensemble. Subsequently, a 2 ns simulation in the constant particle number, pressure, and temperature (NPT) ensemble was carried out at 298 K and 1 atm to obtain equilibrium structures.

The fractional free volume (FFV) was calculated using the Connolly surface method with a probe radius of 1.0 Å according to

$$FFV=\frac{V_{f}}{V_{t}}$$

where *V*ₜ is the total cell volume and *V_f_* is the free volume.

The mean-square end-to-end distance, $\left\langle Ree^{2} \right\rangle=\left( r_{end} - r_{start} \right)^{2}$, was calculated for the polymer chains and averaged over the final 500 ps of the equilibrated trajectories. Values are reported in Å².

**S7. Density functional theory calculations**

Density functional theory (DFT) calculations were performed using Gaussian 16. Geometry optimizations employed B3LYP/6-31G(d) for the PAA models and B3LYP/def2SVPP for all atoms in the silver-containing models. Single-point electronic energies were subsequently calculated at the B3LYP/def2TZVP level using the optimized geometries. Ag was treated using the effective core potentials associated with the def2SVPP and def2TZVP basis sets, replacing 28 core electrons. All calculations were performed in the gas phase, without an implicit solvent model.

The neutral PAA model had the composition C₁₄H₁₁NO₃, whereas the neutral silver-containing model had the composition C₁₄H₁₀AgNO₃. Both were assigned a total charge of zero and a singlet multiplicity. The corresponding deprotonated species were assigned a total charge of −1 and a singlet multiplicity. H⁺ was assigned a charge of +1 and a multiplicity of 1.

The gas-phase deprotonation energy at the amide N–H site was defined as ΔEdeprot = E(A⁻) + E(H⁺) − E(AH), where AH represents the model before deprotonation and A⁻ represents the corresponding species after removal of the proton. The reported values are electronic-energy differences and are distinct from hydrogen-bond interaction energies and homolytic N–H bond dissociation energies.

Each transition state was verified to exhibit a single imaginary frequency corresponding to the intended reaction coordinate. Intrinsic reaction coordinate (IRC) calculations were performed in both directions at the respective geometry-optimization levels, with ten steps calculated in each direction. The trajectories terminated at the specified step limit and describe local reaction paths around the transition states; convergence to endpoint minima was not established by these calculations.

The reaction-energy profiles were constructed from the B3LYP/def2TZVP single-point electronic energies, without zero-point vibrational energy or thermal corrections. Relative energies were referenced to the initial structure of each respective pathway. Each elementary-step barrier was defined as the difference between the transition state and its preceding minimum. Energy differences were converted using 1 Hartree = 627.509474 kcal mol⁻¹.

The DFT models do not include siloxane segments and are not intended to represent the complete molecular structure of the es-PI-0.5 precursor.

**S8. Resistance to repeated tape removal**

Resistance to repeated tape removal was evaluated using 3M 600P tape. A fresh piece of tape was applied to fully cover the patterned silver layer, pressed onto the surface manually, and left in contact for 1 min before being slowly peeled off by hand. The same patterned region was tested repeatedly for 20 removal cycles, with fresh tape used for each cycle. The peeling angle and speed were not quantitatively controlled or recorded. The electrical response was measured at three locations on the same specimen (zone 1–3 in Figure 4c). This qualitative assessment evaluates resistance to repeated tape removal under the stated conditions and does not quantify interfacial adhesion strength.

**Supplementary Notes**

**Supplementary Note 1. Molecular design of self-adaptive stretchable es-PI**

Conventional fully aromatic polyimides exhibit outstanding thermal stability and mechanical strength but are intrinsically brittle because of their rigid backbone structures and limited chain mobility. To overcome this limitation, aminopropyl-terminated siloxane segments were incorporated into the BPDA/ODA polyimide backbone through copolymerization. Unlike simple blending, the siloxane component is covalently integrated into the polymer network, providing flexible spacers while maintaining the integrity of the imide backbone.

The siloxane segments possess highly flexible Si-O-Si bonds with low rotational energy barriers, allowing polymer chains to undergo reversible conformational rearrangement under external deformation. Upon tensile loading, the siloxane-rich soft segments preferentially accommodate the applied strain through bond rotation and chain extension, whereas the aromatic imide domains preserve the structural framework of the polymer. This cooperative deformation mechanism enables the material to sustain large strains while retaining sufficient mechanical strength.

The molecular dynamics results in Figure 5 show an increase in fractional free volume and a decrease in mean-square end-to-end distance upon siloxane incorporation. These findings indicate altered packing and more compact equilibrium conformations in the simulated chains, consistent with increased scope for conformational rearrangement during stretching.

**Supplementary Note 2. Mechanism of confined-patterned in-situ silver growth**

The confined-patterned in-situ metallization strategy uses the ion-exchange capability of the polyamic acid (PAA) precursor before thermal imidization. During immersion in AgNO₃ solution, Ag⁺ ions coordinate with carboxylate groups in the patterned PAA regions. The predefined distribution of the precursor provides a basis for spatially selective silver formation during subsequent processing.

After ion exchange, treatment with dimethylamine borane reduces the incorporated Ag⁺ to silver nanoparticles. The scanning electron microscopy (SEM) images in Figure 3b show particle formation on the patterned precursor after reduction and an increase in particle size and connectivity after thermal imidization. These observations support the formation and consolidation of the surface silver network during the metallization sequence.

Thermal imidization converts the PAA precursor into polyimide and promotes the growth and coalescence of silver nanoparticles. The DFT calculations indicate that silver incorporation alters the electronic structure of the precursor model and reduces the first-step electronic-energy barrier along the calculated imidization pathway. These results provide insight into the local cyclization energetics, while the energy profile of the complete pathway must also be considered. The calculations do not independently establish the kinetics of the experimental thermal-imidization process or silver nanoparticle consolidation.

The coupled ion-exchange, reduction, and imidization process enables conductive silver patterns to be generated directly within predefined regions without additional adhesion layers or post-patterning transfer. The tape-removal and resistance measurements in Figure 4b,c support retention of an electrically connected silver network during repeated tape removal under the reported conditions. Partial embedding of silver particles and associated mechanical interlocking are proposed as possible contributors to interfacial anchoring, as illustrated schematically in Figure 4a. The silver depth distribution and embedding depth were not directly determined, so the contribution of this proposed configuration to adhesion remains to be established. The atomic force microscopy (AFM) results in Figure 3e describe the exposed surface topography and do not resolve the buried metal–polymer interface.

**Supplementary Note 3. LC resonance and smartphone-assisted near-field excitation**

The device-level demonstrations examine two aspects of the es-PI-0.5@Ag platform: its resonance response under deformation and its use in smartphone-assisted near-field excitation. The strain-dependent S₁₁ spectra were measured using a PNA-N5244A network analyzer while the device underwent overall tensile deformation. The resonance frequency decreased from 13.59 MHz at 0% strain to 11.20 MHz at 40% strain, demonstrating a measurable response to the applied deformation.

In an ideal lumped LC description, the resonance frequency is related to the effective inductance L and capacitance C by

$$f₀ = 1/(2\pi\surd(LC))$$

Changes in these effective electrical parameters during deformation can therefore contribute to the observed frequency shift. However, the individual contributions of the patterned coil and the PEDOT:PSS-containing component were not independently resolved. The accompanying broadening and shallowing of the resonance dip may involve deformation-induced resistive losses, but the quality factor and individual loss contributions were not quantified.

In a separate demonstration, a Redmi K70 smartphone with NFC activated provided near-field excitation to a circuit incorporating the patterned silver coil, a BAT54 Schottky diode, PEDOT:PSS, and a fixed capacitor. The rectified DC output was detected using a multimeter at a phone–device separation of approximately 0.9 cm. The smartphone application was used only to activate NFC operation; it did not receive or display strain, resonance-frequency, or other circuit data. Numerical output-voltage values were not recorded, and the tested separation does not establish a maximum operating range.

These experiments provide preliminary evidence of strain-dependent resonance and smartphone-assisted near-field excitation with rectified output. They do not establish quantitative smartphone-based strain readout or continuous wireless motion monitoring. The network-analyzer connection geometry was not verified, so the S₁₁ measurements are not identified here as validated contactless wireless interrogation.

**Supplementary Note 4. Selection of the optimal siloxane content**

Although es-PI-0.8 exhibited a high estimated nominal elongation at break of 547.49 ± 59.26%, its tensile strength decreased to 3.65 ± 0.34 MPa, limiting practical device handling. In comparison, es-PI-0.5 combined a tensile strength of 25.10 ± 0.78 MPa with an estimated nominal elongation at break of 95.61 ± 3.71%, while retaining a 5% weight-loss temperature of 445 °C. Considering the balance among mechanical strength, deformability and thermal stability, es-PI-0.5 was selected for subsequent metallization and device fabrication.

**Supplementary Figures**

**Figure S1. Engineering stress–strain curves of pristine BPDA/ODA-type PI and es-PI films with different siloxane contents. Curves 1–5 represent five independent specimens for each composition. The strain coordinate of each curve was shifted by subtracting its first recorded strain, while retaining the original initial grip separation as the denominator. This adjustment provides an approximate strain reference and does not constitute a validated correction for initial slack. Details of the calculation and its limitations are provided in Supplementary Method S4.**

**Figure S2. Thermogravimetric analysis (TGA) curves of esPI with different siloxane contents and BPDA/ODA-type PI.**

**Figure S3. Storage modulus and Tan(delta) curves for esPI with different siloxane contents compared to pure BPDA/ODA-type PI.**

**Figure S4. Coefficient of thermal expansion (CTE) curves for esPI with different siloxane contents compared to pure BPDA/ODA-type PI.**

**Figure S5. Comparison of the tensile strength and elongation at break of esPI with different siloxane contents versus pure BPDA/ODA-based materials after 8 hours of** **ultraviolet (UV) irradiation at 60°C.**

**Figure S6. Comparison of Infrared Absorbance Between esPI with Different Siloxane Contents and Pure BPDA/ODA Types After 8 Hours of UV Irradiation at 60°C.**

**Figure S7. Changes in the mass and dimensions of esPI-0.5 after immersion in a human sweat simulant.**

**Figure S8. Comparison of UV transmittance and actual transmittance between esPI-0.5 and BPDA/ODA-type PI.**

**Figure S9. Comparison of Water Contact Angles for esPI with Different Siloxane Contents.**

**Figure S10. Force-displacement curve for nanoindentation of esPI-0.5.**

**Figure S11. Infrared Absorbance of the esPI Precursor (PAA) Before and After Ion Exchange.**

**Figure S12-1. X-ray photoelectron spectroscopy (XPS) survey spectra of the PAA precursor before and after Ag⁺ ion exchange, prior to chemical reduction.**

**Figure S12-2. High-resolution Ag 3d X-ray photoelectron spectroscopy (XPS) spectrum of the final es-PI-0.5@Ag film after chemical reduction and thermal imidization.**

**Figure S13. SEM images of the esPI precursor (PAA) following imidization after ion exchange for varying durations.**

**Figure S14. Changes in the water contact angle of esPI precursors (PAA) following imidization after ion exchange for varying durations.**

**Figure S15. Normalized resistance change (ΔR/R₀) versus nominal strain for selected stretching–releasing cycles (1, 2, 5, 10, 25, and 50) of es-PI-0.5@Ag during the test shown in Figure 4f, with a maximum strain of 50%. Here, ΔR = R − R₀, and R₀ = 8.9 Ω is the resistance before the first cycle, used as the common reference for all displayed cycles.**

**Figure S16. SEM images of es-PI-0.5@Ag before (left) and after (right) 50 stretching–releasing cycles at a maximum nominal strain of 50%. The post-cycling images were obtained from the specimen used for the electrical test in Figure 4f. Insets show higher-magnification views.**

**Figure S17. Schematic comparison of the solubility of PAA and PAA-Ag⁺ in N,N-dimethylformamide (DMF).**

Figure S18. Forward and reverse intrinsic reaction coordinate (IRC) profiles initiated from the transition states of the PAA and silver-containing models: (a) PAA TS1, (b) PAA TS2, (c) silver-containing TS1, and (d) silver-containing TS2. Calculations were performed at B3LYP/6-31G(d) for PAA and B3LYP/def2SVPP for the silver-containing models. Energies are referenced to the starting transition-state structure of each profile. Each trajectory comprises ten steps in each direction and terminated at the specified step limit; convergence to endpoint minima was not established by these calculations.

**Supplementary Tables**

**Supplementary Table 1**

| **Sample** | **BPDA [mmol]** | **ODA**  **[mmol]** | **PDMS[mmol]** | **Solid content [wt%]** |
| --- | --- | --- | --- | --- |
| PI | 4.72 | 4.72 | 0 | 12 |
| es-PI-0.1 | 8.23 | 7.40 | 0.83 | 20 |
| es-PI-0.2 | 7.22 | 5.78 | 1.44 | 20 |
| es-PI-0.5 | 5.28 | 2.64 | 2.64 | 20 |
| es-PI-0.8 | 4.16 | 0.833 | 3.33 | 20 |

Table 1 BPDA: 3,3′,4,4′-biphenyltetracarboxylic dianhydride; ODA: 4,4′-oxydianiline; PDMS: aminopropyl-terminated poly(dimethylsiloxane), with a supplier-reported nominal molecular weight of approximately 1000 g mol⁻¹.

**Supplementary Table 1. Monomer feed compositions and solid contents used to prepare the pristine PI and es-PI films.**

**Supplementary Table 2**

| **Sample** | | **Tensile strength [MPa]** | **Estimated nominal elongation at break [%]** |
| --- | --- | --- | --- |
| PI | 125.90 ± 3.28 | | 9.51 ± 0.40 |
| es-PI-0.1 | 78.57 ± 0.58 | | 18.88 ± 0.31 |
| es-PI-0.2 | 63.26 ± 1.26 | | 57.80 ± 2.59 |
| es-PI-0.5 | 25.10 ± 0.78 | | 95.61 ± 3.71 |
| es-PI-0.8 | 3.65 ± 0.34 | | 547.49 ± 59.26 |

**Supplementary Table 2. Mechanical properties of pristine PI and es-PI films.**

Values are reported as mean ± sample standard deviation (SD) for five independent specimens per composition (n = 5). Tensile strength and elongation were determined separately for each specimen before statistical analysis. The elongation estimate was calculated by subtracting the first recorded strain from the recorded strain immediately before fracture, while retaining the original initial grip separation as the denominator. This procedure assumes that the first recorded point approximates the strain reference after specimen straightening; it is not a validated correction for initial slack. SD describes specimen-to-specimen variability and does not include uncertainty in the assumed strain reference. PI and es-PI denote the unmodified and siloxane-modified polyimide films, respectively. Details are provided in Supplementary Method S4

**Supplementary Table 3**

| **Sample** | **T_d5%_ [°C]** | **Residual weight at 800 °C [%]** | **T_g_ (°C)** | **CTE [ppm K**^-^**¹]** |
| --- | --- | --- | --- | --- |
| PI | 539 | 60.9 | 282.1 | 45.5 |
| es-PI-0.1 | 494 | 54.3 | 252.03 | 52.2 |
| es-PI-0.2 | 465 | 44.8 | 223.14 | 105.4 |
| es-PI-0.5 | 445 | 31.4 | 183.1 | 185.3 |

**Supplementary Table 3. Thermal properties of pristine PI and es-PI films.**

**Supplementary Table 4**

| **Model** | **Structure** | **Electronic energy (Hartree)** | **Relative energy**  **(kcal mol⁻¹)** |
| --- | --- | --- | --- |
| PAA | START | −820.8930383 | 0.00 |
| PAA | TS1 | −820.8103513 | 51.89 |
| PAA | MID | −820.8713269 | 13.62 |
| PAA | TS2 | −820.8130571 | 50.19 |
| PAA | END | −820.8930021 | 0.02 |
| PAA–Ag | START | −967.3362394 | 0.00 |
| PAA–Ag | TS1 | −967.2991423 | 23.28 |
| PAA–Ag | MID | −967.2995756 | 23.01 |
| PAA–Ag | TS2 | −967.2566345 | 49.95 |
| PAA–Ag | END | −967.3235123 | 7.99 |

**Supplementary Table 4. Calculated electronic energies and relative energies along the imidization pathways of the PAA and silver-containing models.**

Electronic energies were calculated in the gas phase at the B3LYP/def2TZVP level using the optimized geometries described in Supplementary Method S7, without zero-point vibrational energy or thermal corrections. Relative energies were referenced to the START structure of each respective pathway and calculated as ΔE = (E_structure_ − E_START_) × 627.509474, with electronic energies in Hartree and ΔE in kcal mol⁻¹. PAA denotes the poly(amic acid) model, and PAA–Ag denotes the silver-containing model. START, TS, MID, and END denote the initial structure, transition state, intermediate, and final structure, respectively.
